# Supplementary material for: Structural modifications in strain-engineered bilayer nickelate thin films
Source: Nature. 2026 Apr 1;653(8113):76–82. doi: 10.1038/s41586-026-10446-2 (PMC13149027; doi:10.1038/s41586-026-10446-2)
Supplement: Supplementary file 1 — This file contains Supplementary Figs. 1–23 and Supplementary Tables 1–5; details on octahedral decomposition and generalization of the 4-octahedra model. [file 41586_2026_10446_MOESM1_ESM.pdf]

nature portfolio

https://doi.org/10.1038/s41586-026-10446-2

Supplementary information

Structural modifications in strain-engineered bilayer nickelate thin films

In the format provided by the authors and unedited

Supplemental Information for Structural modifications in strain-engineered bilayer nickelate thin films

Lopa Bhatt,[1,*] Edgar Abarca Morales,[2] Abigail Y. Jiang,[3,4] Eun Kyo Ko,[5,6] Yi-Feng Zhao,[7] Noah Schnitzer,[8,9] Grace A. Pan,[3] Dan Ferenc Segedin,[3] Yidi Liu,[5,10] Yijun Yu,[5,6] Charles M. Brooks,[3] Antia S. Botana,[7] Harold Y. Hwang,[5,6] Julia A. Mundy,[3,4] David A. Muller,[1,9] and Berit H. Goodge[2,†]

[1] *School of Applied & Engineering Physics, Cornell University, Ithaca, NY 14853, USA*
[2] *Max Planck Institute for Chemical Physics of Solids, 01187 Dresden, Germany*
[3] *Department of Physics, Harvard University, Cambridge, MA 02138, USA*
[4] *John A. Paulson School of Engineering and Applied Sciences, Harvard University, Cambridge, MA 02138, USA*
[5] *Stanford Institute for Materials and Energy Sciences, SLAC National Accelerator Laboratory, Menlo Park, CA 94025, USA*
[6] *Department of Applied Physics, Stanford University, Stanford, CA 94305, USA*
[7] *Department of Physics, Arizona State University, Tempe, AZ 85287, USA*
[8] *Department of Materials Science and Engineering, Cornell University, Ithaca, NY 14853, USA*
[9] *Kavli Institute at Cornell for Nanoscale Science, Cornell University, Ithaca, NY 14853, USA*
[10] *Department of Physics, Stanford University, Stanford, CA 94305, USA*

(Dated: March 11, 2026)

* lb628@cornell.edu

† berit.goodge@cpfs.mpg.de; bhgoodge@cornell.edu

CONTENTS

I Structural parameters derived from experimental data and DFT calculations 4
TABLE S1: Structural parameters for the series of strained $La_3Ni_2O_7$ films experimentally measured via x-ray diffraction (XRD). 4
TABLE S2: Structural parameters for the series of strained $La_3Ni_2O_7$ films experimentally measured via STEM. 5
TABLE S3: Structural parameters for strained $La_3Ni_2O_7$ calculated from DFT relaxations as projected for direct comparison to STEM measurements. 6

II Multislice electron ptychography 7
FIG. S1: Illustration of MEP and comparison of different projections of $La_3Ni_2O_7$. 7
TABLE S4: Typical MEP reconstruction parameters. 8
TABLE S5: Individual MEP reconstruction parameters. 8
FIG. S2: Depth-resolved phase slip in oxygen sublattice. 9
FIG. S3: Representative MEP reconstructions from compressive and tensile strained $La_3Ni_2O_7$. 10

III Defects and secondary phases in $La_3Ni_2O_7$ thin films 11

IV ADF-STEM image analysis of $La_3Ni_2O_7$ thin films 13
FIG. S4: Representative ADF-STEM images of $La_3Ni_2O_7$ thin films 13
FIG. S5: Multiple interface structures in $La_3Ni_2O_7$ on SLAO. 14
FIG. S6: Twinning in tensile strained $La_3Ni_2O_7$ on NGO. 15
FIG. S7: Regions of EELS measurements in $La_3Ni_2O_7$ on SLAO. 15
FIG. S8: Fourier transforms of $La_3Ni_2O_7$ films across varying strain. 16

V X-ray diffraction and RSM of $La_3Ni_2O_7$ thin films 17
FIG. S9: Reciprocal space mapping (RSM) of $La_3Ni_2O_7$ on LAO, NGO, and STO. 18
FIG. S10: XRD of $La_3Ni_2O_7$ films on LAO, NGO, and STO. 19

VI Ni-O angle measurements 20
FIG. S11: Illustration of measured angles. 20
FIG. S12: Reported structures of $La_3Ni_2O_7$ at low pressures. 20
FIG. S13: Reported structures of $La_3Ni_2O_7$ at high pressures. 21
FIG. S14: Histograms of Ni-O-Ni angles. 21
FIG. S15: Separated histograms of O-Ni-Ni angles. 22

VII Importance of time coordination for STEM experiments 23
FIG. S16: EELS and MEP measurements of an $La_3Ni_2O_7$ film on SLAO without time coordination. 23

VIII STEM and XRD measured c-axis lattice spacing 24
FIG. S17: $La_3Ni_2O_7$ film c-axis measured by STEM and XRD. 24

IX Octahedral strain decomposition 25
A Extracting octahedra from $La_3Ni_2O_7$ 25

1 Generalized octahedral parametrization . . . 25
2 Geometric interpretation of the **F**-matrix . . . 25
3 Extracting parameters from a single octahedron . . . 27
4 Obtaining the octahedral parameters . . . 29
FIG. S18: Illustration of site- and layer-separation in the octahedral decomposition. . . . 30
FIG. S19: Complete octahedral parameters for considered structures. . . . 31
B Decomposing the straining process . . . 31
1 Reconstructing the bipartite layers . . . 31
2 Layer distortion decomposition . . . 32
3 Reconstruct $La_3Ni_2O_7$ . . . 32

X Transformation into the local basis **33**

XI Bilayer vs Complete structure **33**
FIG. S20: Comparison of band structures for single bilayers and complete unit cells. . . . 34

XII GGA with and without Hubbard U **34**
FIG. S21: Band structures calculated with Hubbard U. . . . 35
FIG. S22: Band structures calculated without Hubbard U. . . . 36
FIG. S23: Additional projections for the calculated band structures. . . . 37

References **38**

I. STRUCTURAL PARAMETERS DERIVED FROM EXPERIMENTAL DATA AND DFT CALCULATIONS

TABLE S1. Structural parameters for the series of strained $La_3Ni_2O_7$ films experimentally measured via x-ray diffraction (XRD). Experimental in-plane lattice constants and strain values are extracted from film peak centroid fits in reciprocal space maps (RSM). The c-axis spacing is calculated by Nelson-Riley fits to the XRD maps for films on LAO, NGO, and STO. The c-axis spacing for the film on SLAO is measured from the XRD (006) Bragg peak.

Substrate (nominal ε)	SLAO (-2.0%)	LAO (-1.2%)	NGO (+0.7%)	STO (+1.9%)
Experimental strain	-1.6%	-0.9%	+0.6%	+1.8%
In-plane axis (Å)	5.33	5.37	5.45	5.52
c (Å)	20.56	20.66 ± 0.13	20.39 ± 0.06	20.01 ± 0.23
Volume $a \times a \times c$ (Å^3)	584.63	595.97	605.98	609.99

TABLE S2. Structural parameters for the series of strained $La_3Ni_2O_7$ films experimentally measured via STEM. Average lattice spacings and bond angle values are measured via MEP- and ADF-STEM as detailed in the main text, with errors taken as the standard deviation of all measurements.

Substrate (nominal ε)	SLAO (-2.0%)	LAO (-1.2%)	NGO (+0.7%)	STO (+1.9%)
Ni-planar O (O-Ni-Ni) angle, top layer (°)	1.0 ± 1.4	0.7 ± 1.3	-4.2 ± 1.7, 5.5 ± 2.4	-5.3 ± 1.2, 6.5 ± 1.8
Ni-planar O (O-Ni-Ni) angle, bottom layer (°)	-1.6 ± 1.5	-1.2 ± 1.2	-5.2 ± 2.1, 4.2 ± 1.9	-5.9 ± 2.0, 6.3 ± 1.1
Ni-planar O-Ni angle (°)	177.4 ± 3	178.1 ± 3	169.3 ± 4, 188.5 ± 4	167.6 ± 4, 191.6 ± 3
In-plane axis (Å)	5.34 ± 0.05	5.36 ± 0.06	5.43 ± 0.07	5.49 ± 0.08
c (Å)	20.6 ± 0.15	20.54 ± 0.16	20.54 ± 0.13	20.32 ± 0.15
OOP Ni - outer apical O spacing (Å)	2.29 ± 0.05		2.28 ± 0.03	2.25 ± 0.04
OOP Ni - inner apical O spacing (Å)	2.03 ± 0.05		2.02 ± 0.03	1.99 ± 0.03
Volume $a \times a \times c$ (Å^3)	587.42 ± 8.88	590.10 ± 10.41	605.62 ± 11.69	612.45 ± 13.41

TABLE S3. Structural parameters for strained $La_3Ni_2O_7$ calculated from DFT relaxations as projected for direct comparison to STEM measurements. Full crystal structure models are provided (Data availability).

Substrate (nominal ε)	SLAO (-2.0%)	STO (+1.9%)
Projected Ni-planar O (O-Ni-Ni) angle, top layer (°)	1.2	7.2, -7.3
Projected Ni-planar O (O-Ni-Ni) angle, bottom layer (°)	-1.2	-7.2, 7.3
Projected Ni-planar O-Ni angle (°)	177.6	165.6, 165.5
In-plane axis (Å)	5.31	5.52
c (Å)	20.812	19.986
Projected OOP Ni-outer apical O spacing (Å)	2.31	2.14
Projected OOP Ni-inner apical O spacing (Å)	1.97	1.95
Volume $a \times a \times c$ (Å^3)	587.2	609.5

II. MULTISLICE ELECTRON PTYCHOGRAPHY

As in high-resolution STEM imaging, a converged probe of high-energy electrons is rastered across the sample (Supplemental Fig. S1). Compared to conventional imaging modes which record only a single intensity at each probe position, a full diffraction pattern is recorded at every real-space position using a high-dynamic range pixel array detector [1]. The phase information of the scattered electrons encoded in the overlapping diffraction disks is used to reconstruct the sample potential through iterative algorithms [1, 2].

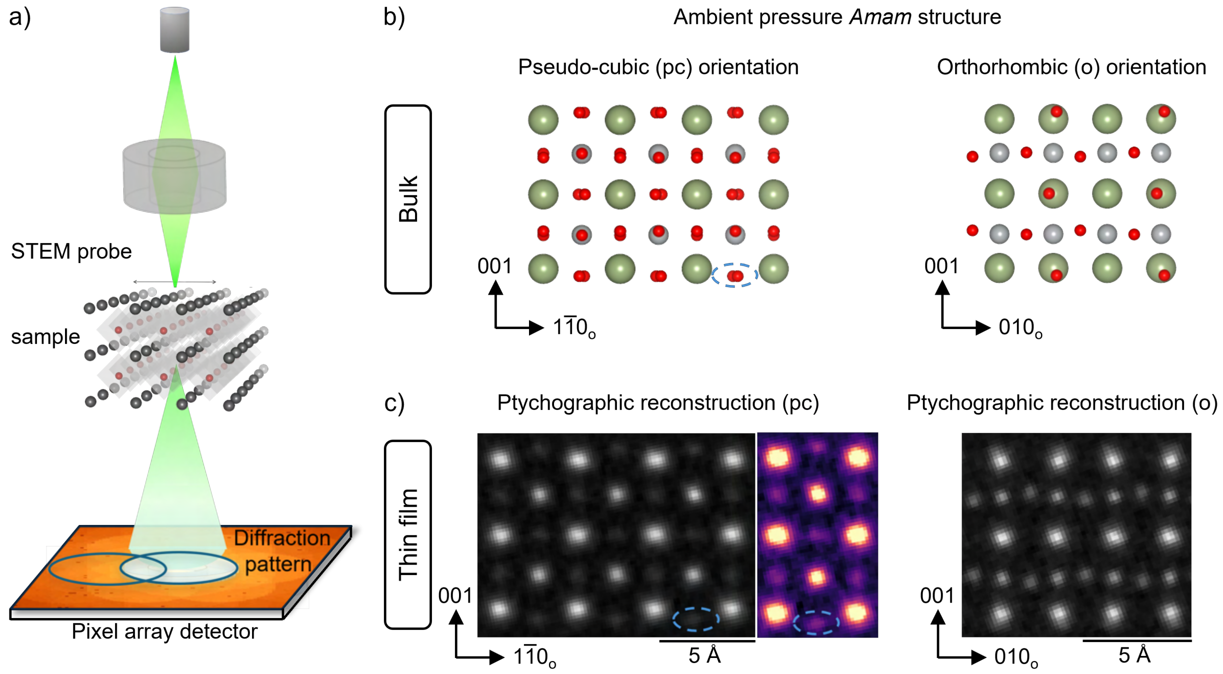

FIG. S1. **a)** Illustration of the experimental setup for multislice electron ptychography (MEP) showing a convergent STEM probe rastering across the sample. The scattered electron diffraction patterns are collected on a high dynamic range pixel array detector [3]. **b)** Experimental bulk structure of a single bilayer as reported by [4] along the pseudo-cubic $[110]_o$ (pc, left) and orthorhombic $[100]_o$ (o, right) projections. The oxygens are anti-phase stacked along the pseudo-cubic orientation, as highlighted by the blue circle [5]. **c)** Experimental multislice electron ptychographic reconstruction of an $La_3Ni_2O_7$ thin film on STO along both pseudo-cubic (left) and orthorhombic (right) projections. A slight elongation of the oxygen columns due to the anti-phase stacking is visible along the pc orientation. The antiphase stacking also leads to a reduction in the intensity of oxygen columns compared to La and Ni columns. A saturated and cropped magma-colored field-of-view of the reconstruction is given for clear visibility of oxygen columns along the pc projection.

TABLE S4. Typical range of values for MEP reconstruction (via fold-slice package [2, 6, 7]) parameters individually optimised for all datasets analysed in this manuscript. Z regularization is the interlayer regularization introduced in [2].

Parameters	value
Accelerating voltage	300 kV
Probe defocus	-50 Å: -190 Å
Convergence angle	26.3 mrad : 30.0 mrad
Scan step size	0.43 Å: 0.63 Å
Slice thickness	6.0 Å: 9.0 Å
Z regularization	0.7 : 0.8
Outer collection angle	55 mrad : 66 mrad
Number of scan positions	256x256
Detector pixels	128x128
Number of probe modes	8
Per pixel dwell time	100 µs

TABLE S5. Values for MEP reconstruction (via fold-slice package [2, 6, 7]) parameters individually optimized.

Parameters	Fig 2a	Fig 2b	Fig 2c	Fig 2d	Fig E1	Fig E2	Fig E6
Accelerating voltage (kV)	300	300	300	300	300	300	300
Probe defocus (Å)	-81	-97	-75	-177	-144	-70	-94
Convergence angle (mrad)	27.6	27.3	27.4	27.8	27.5	28.0	27.4
Scan step size (Å)	0.44	0.43	0.43	0.43	0.43	0.41	0.43

Note that different optimization parameters within each reconstruction, such as convergence angle and step size are interlinked; the variation in value for convergence angle shown here are within a typical spread previously reported [8, 9].

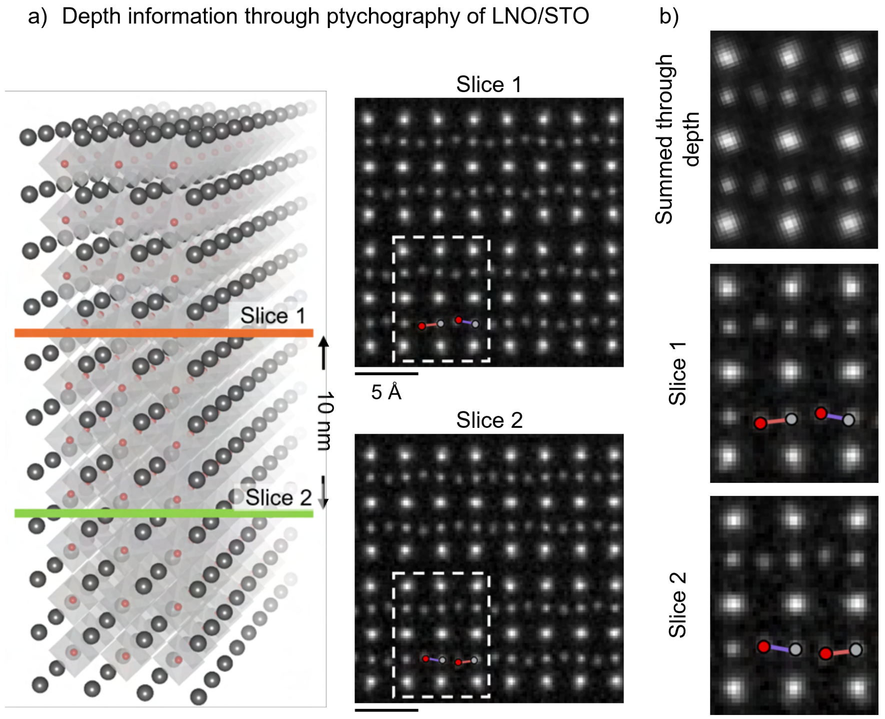

FIG. S2. a) (Left) Schematic of depth information provided through multislice electron ptychography. (Right) Two depth slices separated by $\sim$ 10 nm obtained from the same ptychographic reconstruction of $La_3Ni_2O_7$ /STO. Areas highlighted by white squares are enlarged in b) showing opposite displacements of oxygen columns. When averaged through the total lamella depth (top), the oxygen column position can no longer be precisely identified and the difference between the two regions is lost.

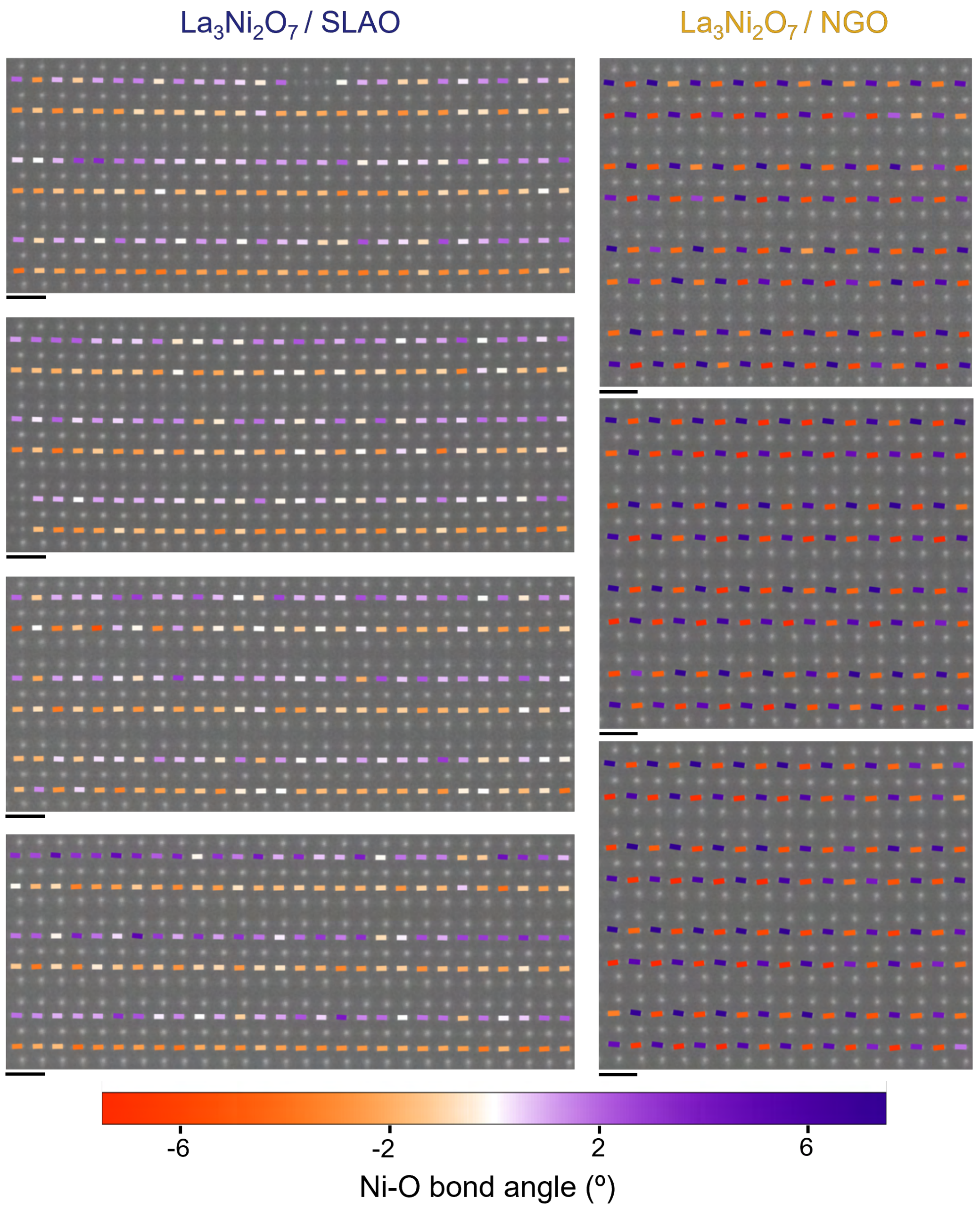

FIG. S3. Multislice electron ptychography reconstructions from randomly chosen regions of $La_3Ni_2O_7$ grown on SLAO (left) and NGO (right) showing agreement between the datasets. Scale bar is 5 Å.

III. DEFECTS AND SECONDARY PHASES IN $La_3Ni_2O_7$ THIN FILMS

Films grown on SLAO, LAO, and NGO yield high-quality crystalline Ruddlesden-Popper nickelates with intermittent mixed-phase intergrowth layers – mainly of La_2NiO_4 – and extended defects spanning the films (Supplemental Figs. S4, 9, 10). Films grown on STO show the highest density of extended defects due to the high tensile strain imposed by the substrate [10, 11]. The lower symmetry structure of tensile-strained $La_3Ni_2O_7$ films show in-plane twinning (Supplemental Fig. S6); all bond angle analysis performed here for films on NGO and STO includes only the *a*-axis projection while avoiding any *b*-axis twins. In tensile strained film on STO, an out-of-phase boundary in the oxygen sublattice through the projection depth of the sample is observed (Supplemental Fig. S2).

While the lattice parameters in Figs. 2 and 3 and the structures used for DFT calculations in Fig. 4 represent an ensemble understanding of the film structure, epitaxial relaxation and crystalline defects can locally modify these parameters such that they may fall outside the window required for superconductivity, hindering the global superconducting transition. Extended Data Fig. 6a shows a large field-of-view ADF-STEM image of the superconducting $La_3Ni_2O_7$ thin film on SLAO. Near strain-relieving dislocations marked by yellow $\top$, the IP La-La distances measured by quantitative atom-tracking (Extended Data Fig. 6b) show clear expansion away from the average compressed value of $\sim$2.67 Å. Other signs of minor relaxation can also be observed by the corresponding reduction of the OOP La-La distances near the surface of the film (Extended Data Fig. 6c). A second crystalline defect which extends throughout the vertical direction of the film can be traced to a step-edge in the SLAO substrate (Supplemental Fig. S5) and correlates with stacking variation on either side. A variety of different interfacial atomic structures are observed by microscopic studies here and elsewhere [12–15], and thin films grown on LAO, NGO, and STO under hydrostatic pressure have also shown superconducting transitions [16]. Superconductivity therefore appears to be largely interface-independent, though more subtle effects remain to be explored [17, 18].

Single layers of the $n = 1$ Ruddlesden-Popper phase La_2NiO_4 are especially visible by their reduced OOP La-La spacing (Extended Data Fig. 6c) and are observed in all films within our series (Supplemental Fig. S4). Similar intergrowths have also been documented in bulk crystals of $La_3Ni_2O_7$ [19–22], though the importance for superconductivity of such polymorphs along the vertical stacking direction has not been established. While there has

been mounting evidence to suggest bulk-like superconductivity hosted in the pristine bilayer regions of the thin films [13], we can not rule out the possibility of filamentary superconductivity in the defect regions. When different intergrowths meet laterally in the film, however, the large internal crystalline strain can lead to significant local modification of the lattice constants, as visible in several regions of Extended Data Fig. 6b,c. We speculate that these regions of concentrated internal strain may have a stronger impact on the macroscopic properties of the very thin films studied here, while similar effects may be more dilute in bulk sample volumes. These observations also provide a tantalizing hint towards the possibility of engineering filamentary superconductivity at highly strained heterostructure interfaces or grain boundaries in bulk crystals.

In addition to crystalline defects and stacking variation, more subtle point-like disorder such as atomic vacancies may also have a strong impact on superconductivity. Previous studies in both bulk and thin film $La_3Ni_2O_7$ established the importance of oxygen stoichiometry: bulk samples which are oxygen-deficient exhibit insulating behavior [23–25], and thin films on SLAO require additional post-growth ozone annealing to exhibit a superconducting transition [12, 26]. Investigating oxygen occupancy at mesoscopic length scales (Extended Data Fig. 6) via O-K edge electron energy loss spectroscopy (EELS) shows variation in oxygen stoichiometry within a single $La_3Ni_2O_7$ sample [5, 27, 28]. Extended Data Fig. 6d shows two EEL spectra collected on the same day from two highly crystalline regions of the same $La_3Ni_2O_7$ thin film on SLAO (Supplemental Fig. S7). One region (black) shows a pronounced pre-peak in the O-K edge around 528 eV, consistent with previous measurements of near-stoichiometric $La_3Ni_2O_7$ which is plotted for reference as a dotted pink line [5]. In a second region (gray) the pre-peak is absent suggesting a reduced oxygen occupation [5, 28]. It is likely that concentrated oxygen vacancies and proximity to defects may also modify the local octahedral coordination as seen in Extended Data Fig. 6e showing local variation of the Ni-O bond angle. Future studies correlating precise structural measurements with oxygen stoichiometry and electronic transport may provide further insights in this regard. Rare earth substitution by Pr has also demonstrated improvement in the superconducting transition [26, 29, 30] and critical current density J_c [13], though whether these improvements are driven by differences in ionic radii or thermodynamic stability remains to be established.

IV. ADF-STEM IMAGE ANALYSIS OF $La_3Ni_2O_7$ THIN FILMS

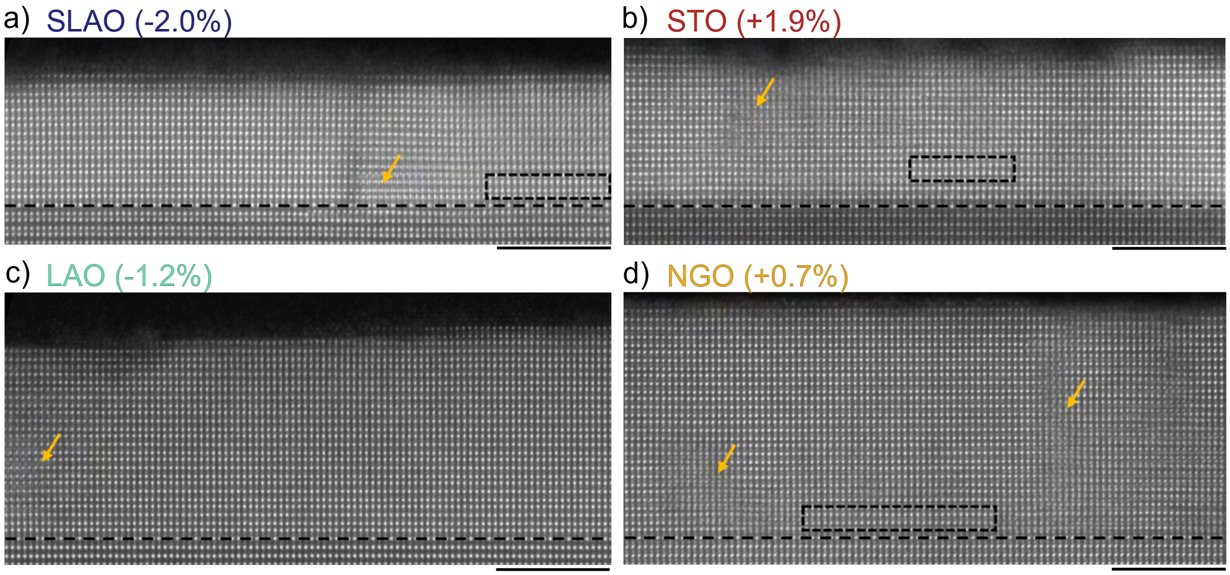

FIG. S4. **a-d)** Representative ADF-STEM images of $La_3Ni_2O_7$ thin films grown on SLAO (a), STO (b), LAO (c), and NGO (d). Black dashed lines mark the approximate position of the interface. Structural defects are indicated by yellow arrows and intergrowths of La_2NiO_4 by black boxes. Scale bar is 5 nm.

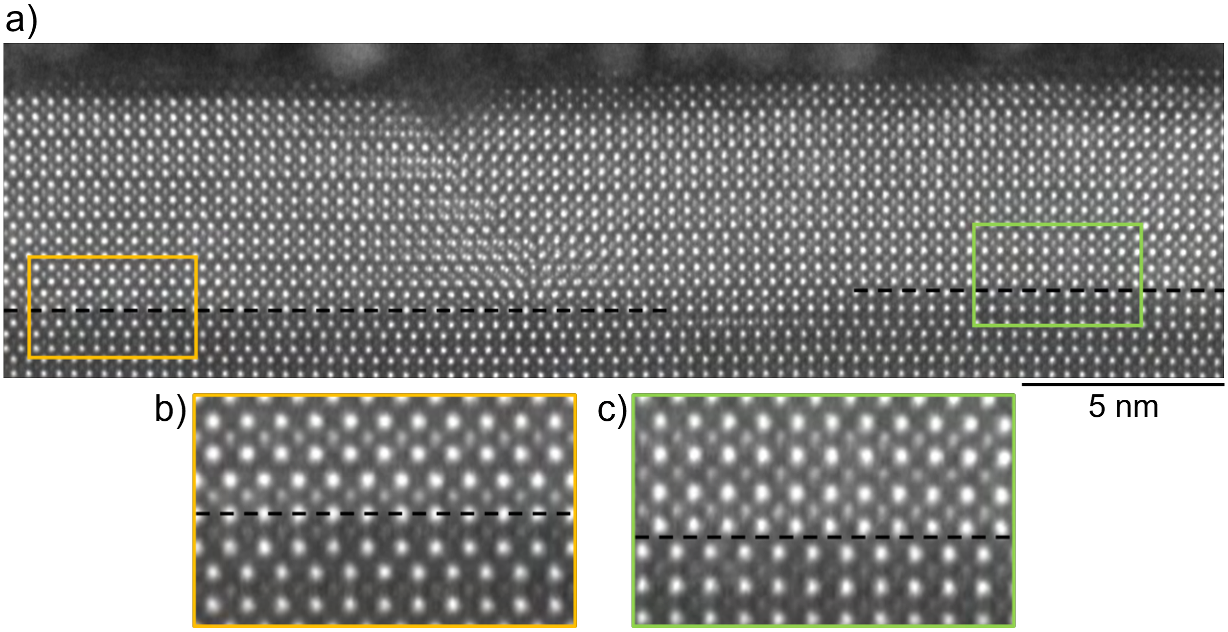

FIG. S5. **a)** ADF-STEM image of $La_3Ni_2O_7$ film grown on SLAO along the pseudo-cubic $[110]_o$ projection. Black dashed line indicates the approximate position of interface inferred from changes in the intensity of atomic columns. The interfacial structure varies from the left side of the image to the right with a step edge in the middle. The two distinct interfacial structures are highlighted with yellow and green boxes. **b,c)** Enlarged view of the interfaces in yellow (b) and green (c) boxes. In (b), the interface between substrate and film appears in the middle of the bilayer followed by a single layer of La_2NiO_4 while in (c) there is a Ruddlesden-Popper spacer layer gap at the interface and the first layer of the film forms as trilayer $La_4Ni_3O_{10}$.

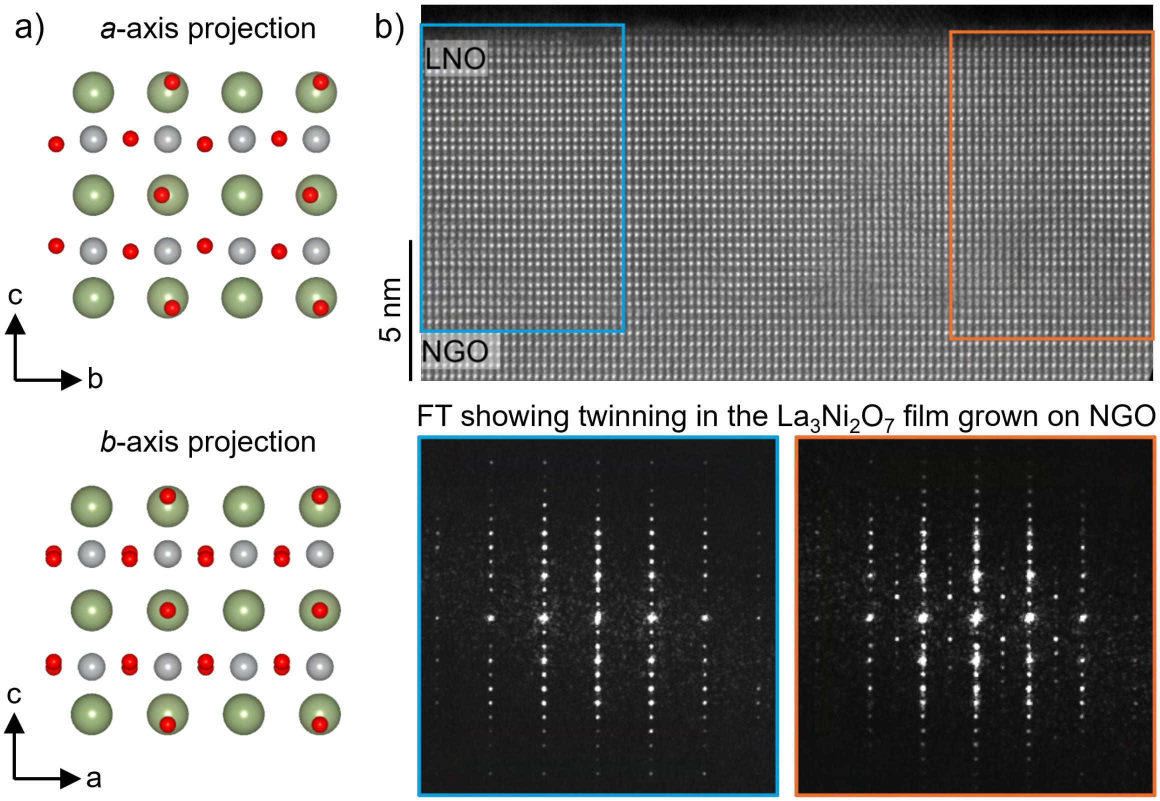

FIG. S6. **a)** Experimental bulk structure of a single bilayer as reported by [4] along the a-axis projection (top) and b-axis projection (bottom). **b)** ADF-STEM image (top) of $La_3Ni_2O_7$ grown on NGO. Fourier transforms (FTs) of two different areas of the film marked by blue and orange boxes show distinct peaks. The orange region has half-order peaks that are lacking in the blue region which are consistent with the inequivalent a (orange) and b (blue) projections of the *Amam*-like structure, indicating the presence of twinning in the film. Measurements of Ni-planar O bond angles by MEP in the main text is limited to regions with a-axis projection.

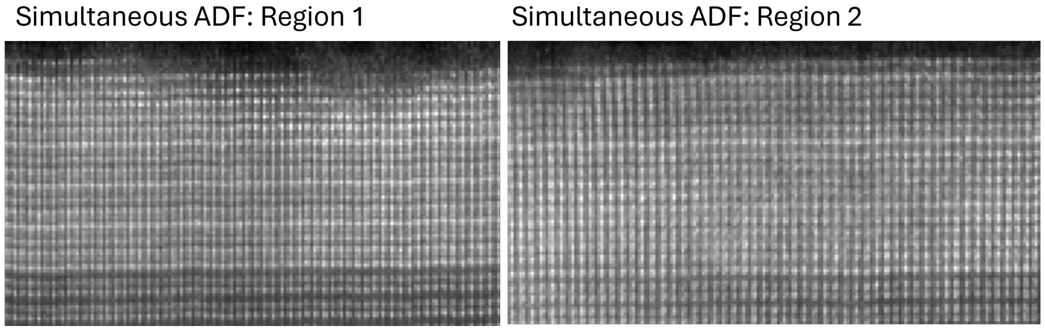

FIG. S7. ADF images of Region 1 and Region 2 acquired simultaneously with the EEL spectra shown in Extended Data Fig. 6. Both ADF images confirm the overall crystalline quality and majority adherence to $La_3Ni_2O_7$ stacking. Note that the reduced signal-to-noise ratio of the images is due to the low probe currents used for EELS measurements and apparent "distortions" to the lattice are from small amounts of sample drift during the long acquisition times.

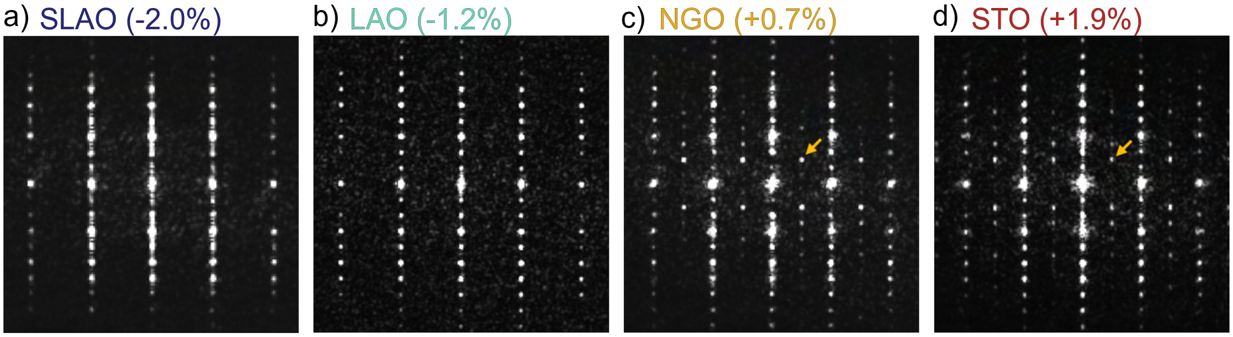

FIG. S8. **a-d)** Fourier transforms (FTs) of ADF-STEM images of $La_3Ni_2O_7$ films grown on SLAO (a), LAO (b), NGO (c), and STO (d). Extra peaks (yellow arrow) are visible in the tensile strained films and absent in the compressive films indicating structural symmetries consistent with $Amam$ and $Fmmm$ or $I4/mmm$, respectively.

V. X-RAY DIFFRACTION AND RSM OF $La_3Ni_2O_7$ THIN FILMS

As shown in Extended Data Fig. E6, the thin films involve areas with relaxation and defects that inherently scatter differently compared to the pristine regions of the film. Therefore, it is crucial to account for the various constructive and destructive interference occurring due to defects as well as the pristine bilayer through extensive simulation for quantitative interpretation of reciprocal space mapping (RSM) experiments (Supplemental Fig. S9). Here, we present a rough estimate, not a quantitative one, of the strain heterogeneity present in the thin films through RSM analysis.

We performed a simple intensity analysis to estimate the fraction of coherently strained versus partially relaxed regions for superconducting $La_3Ni_2O_7$ /SLAO using RSM given in [12]. Because the estimate depends on the metric used, we considered two approaches. Using the peak heights of the corresponding diffraction features yields an estimate that approximately 78% of the film remains coherently strained, while using the integrated peak areas gives a smaller value of about 18%. These two approaches therefore suggest a plausible range for the coherently strained fraction of roughly 20–80% in the superconducting film. While this estimate should be regarded as an order-of-magnitude estimation, it indicates that a substantial portion of the film is coherently strained. Similar analysis performed on RSM data provided in Supplemental Fig. S9 yields a smaller relaxation fraction for films on NGO and STO.

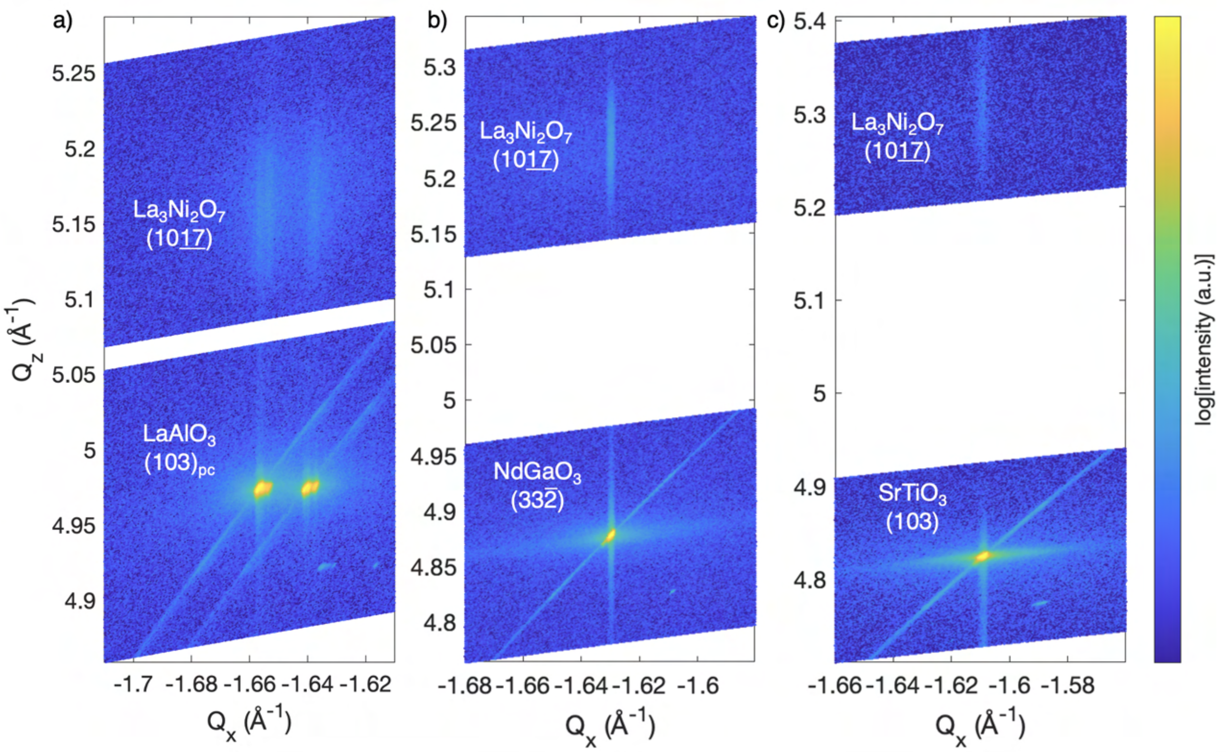

FIG. S9. Reciprocal space maps (RSM) of $La_3Ni_2O_7$ thin films with the pseudo-tetragonal (10$\underline{17}$) Bragg peaks of $La_3Ni_2O_7$ alongside the following substrate peaks: **a)** $(103)_{pc}$ pseudo-cubic peak of LAO(100), **b)** (33$\bar{2}$) peak of NGO(110), and **c)** (103) peak of STO(001). In-plane lattice constants are extracted from the $La_3Ni_2O_7$ film peaks. RSM of $La_3Ni_2O_7$ on SLAO(001) is shown in extended data of [12]. The LAO(100) sample exhibits multiple substrate peaks (and thus multiple signals from the strained $La_3Ni_2O_7$) due to twinning in the substrate, and the left-most peak displayed was used to extract the in-plane parameter based on the RSM measurement alignment.

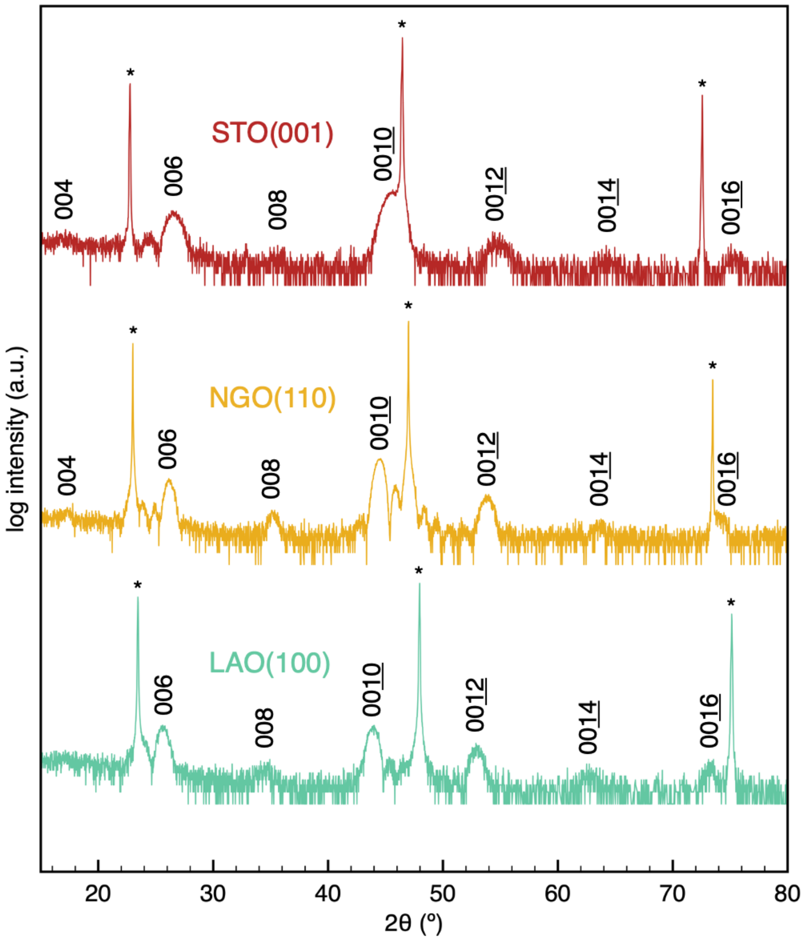

FIG. S10. X-ray diffraction (XRD) of $La_3Ni_2O_7$ films on LAO(100), NGO(110), and STO(001) substrates. Substrate peaks are labeled with *. A Nelson-Riley fit of the indexed (00l) peaks of $La_3Ni_2O_7$ was used to extract the c-axis lattice parameters and corresponding error as measured by XRD [31]. XRD of $La_3Ni_2O_7$ on SLAO(001) is shown in Figure 1 of [12].

VI. NI-O ANGLE MEASUREMENTS

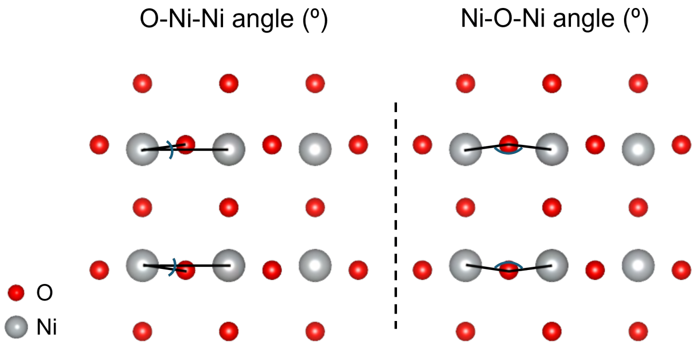

FIG. S11. Illustration of the measured O-Ni-Ni (left) and Ni-O-Ni (right) angles.

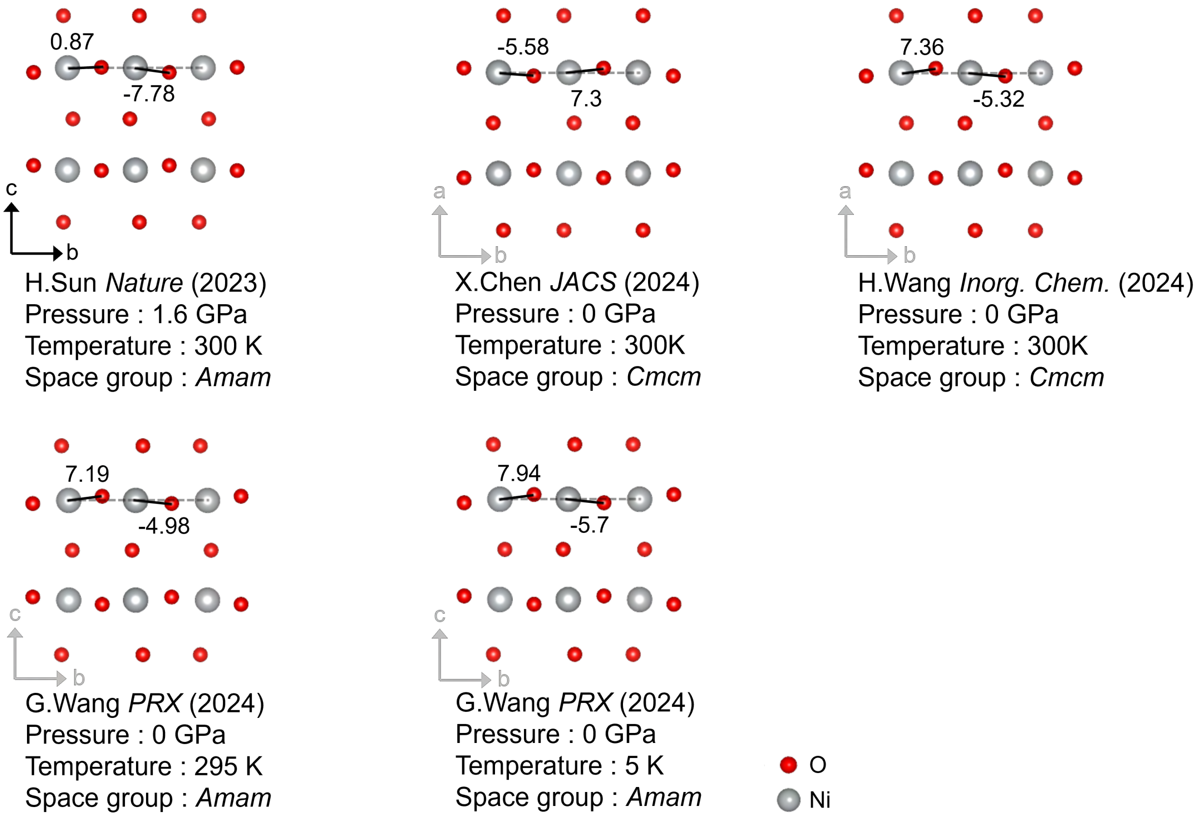

FIG. S12. Various reported structures of $La_3Ni_2O_7$ at low pressure showing a single bilayer of the Ni-O_6 octahedra (La sites are omitted for clarity), showing variability in the assigned space groups and approximate measured Ni-planar O bond angles. Structures reported by [4, 20, 32, 33].

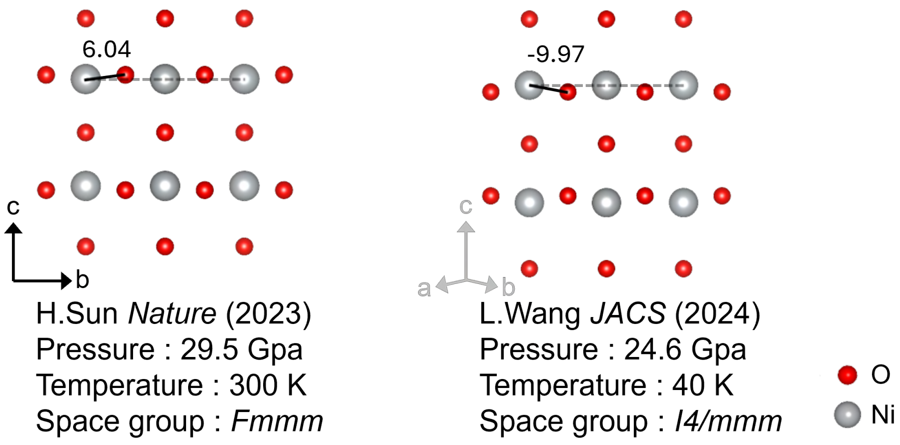

FIG. S13. Two reported structures of $La_3Ni_2O_7$ at high-pressure and different temperatures, here showing only a single bilayer of the Ni-O_6 octahedra (La sites are omitted for clarity). Approximate Ni-planar O bond angles are noted. Beyond their space group assignments ($Fmmm$ and $I4/mmm$), the structures also differ by the distortion direction of the planar O either away from (left) or towards (right) the center plane of the bilayer. Structures reported by [4, 34].

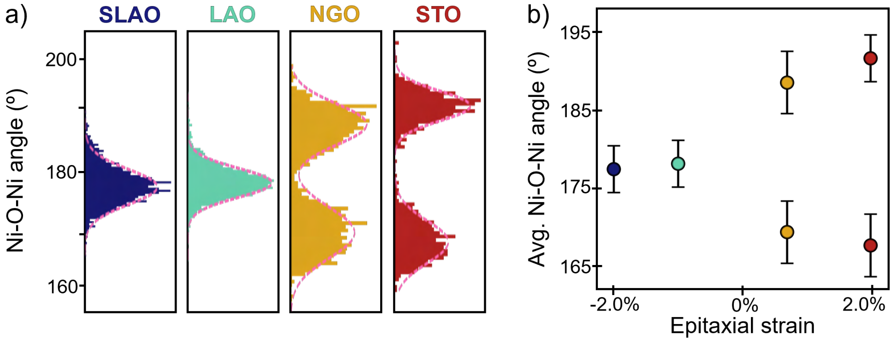

FIG. S14. a) Ni-O-Ni angles of all four thin films corresponding to the schematic at the right of Fig. S11. The pink dashed line is a Gaussian fit to the angle distribution. b) Average Ni-O-Ni angle for films under different epitaxial strains

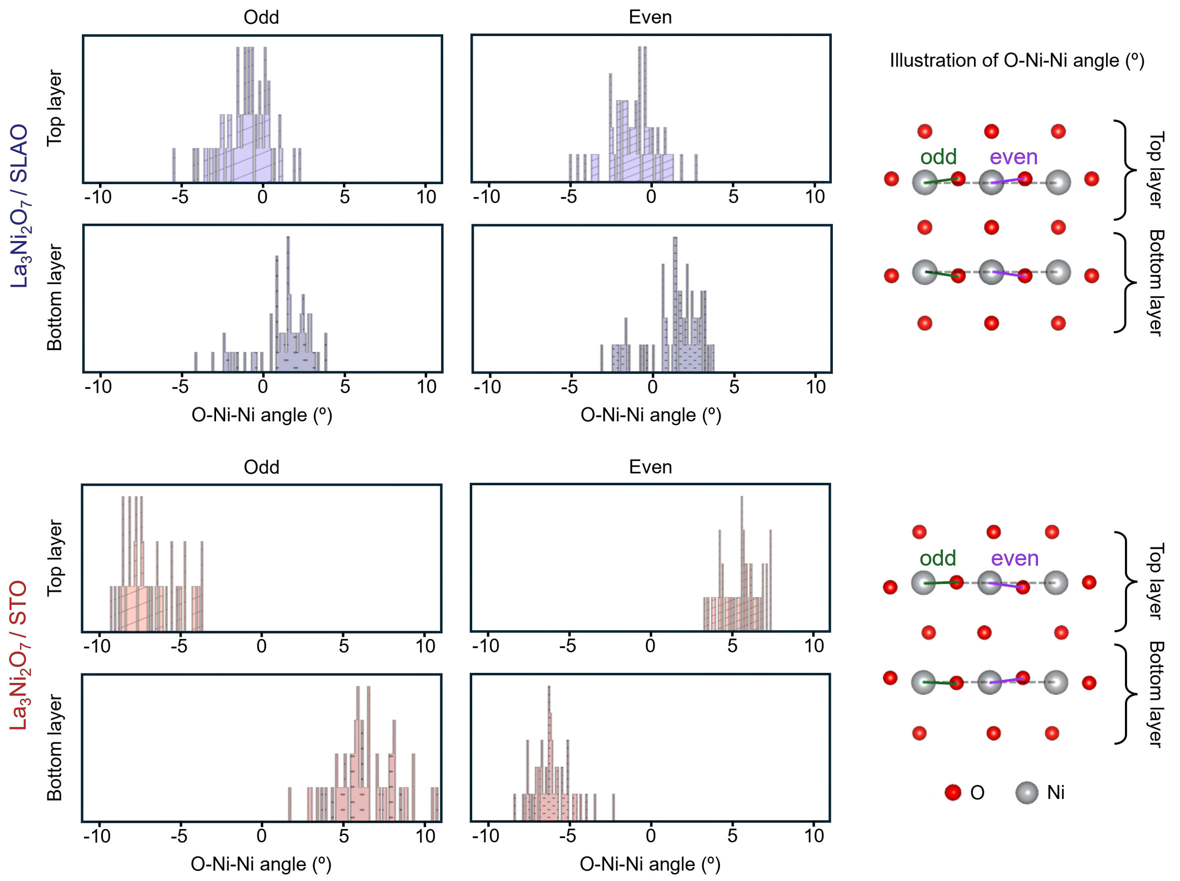

FIG. S15. Histograms of upper and lower NiO_2 plane O-Ni-Ni angles separated between odd and even sites measured via MEP for $La_3Ni_2O_7$ thin films grown on SLAO (top panel) and STO (bottom panel). As discussed in Figure 2 of the main text, the compressively strained films exhibit distributions for the bottom and top layer distortions which can be captured by a single Gaussian, while the tensile-strained films require two-Gaussian fits to fully capture the positive and negative distortions in each layer.

VII. IMPORTANCE OF TIME COORDINATION FOR STEM EXPERIMENTS

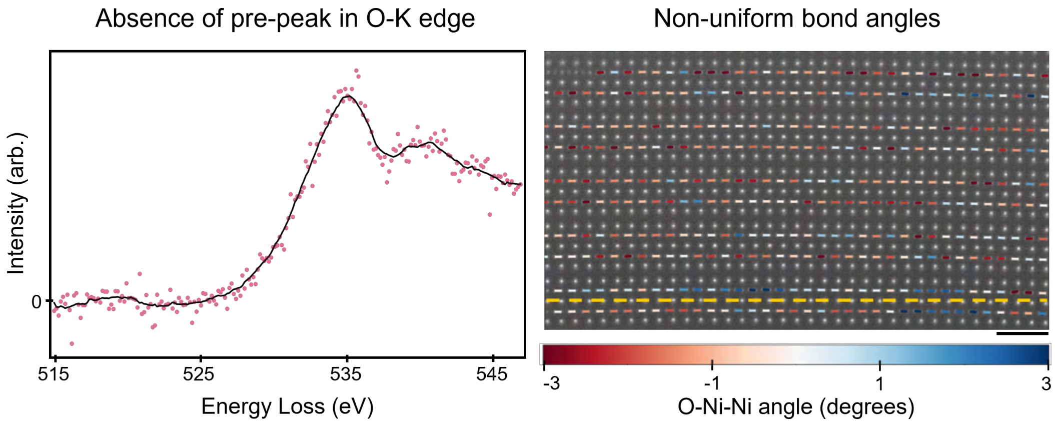

FIG. S16. O-K edge (left) and planar O-Ni-Ni angle maps (right) measured on $La_3Ni_2O_7$ thin film on SLAO without careful time-coordination.

VIII. STEM AND XRD MEASURED c-AXIS LATTICE SPACING

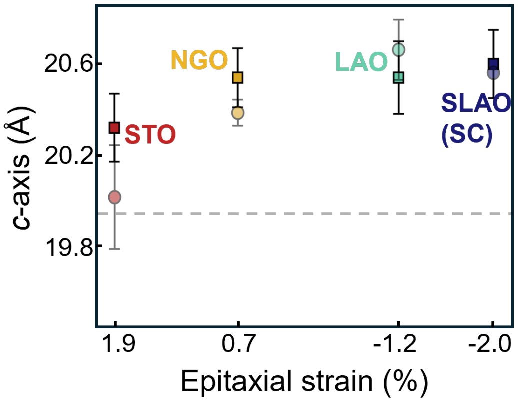

FIG. S17. c-axis lattice constants of $La_3Ni_2O_7$ thin films as a function of nominal epitaxial strain measured via XRD (circles) and ADF-STEM (squares). Errors from Nelson-Riley fits of the XRD data for films on STO, NGO, and LAO are plotted along with the standard deviation of the ADF-STEM measurements. XRD and STEM measurements of c-axis lattice spacing agree within the uncertainty of the measurements.

IX. OCTAHEDRAL STRAIN DECOMPOSITION

While experimentally disentangling contributions from different structural changes is challenging or impossible due to strong coupling between distortions, the octahedral decomposition approach allows us to separate the total strain in epitaxial films or under hydrostatic pressure into virtual structures subject to particular distortions in isolation, and to track their individual effects on the electronic structure. This section explains the strain decompositions shown in the main text in a step-by-step fashion. For simplicity, here we focus on the comparison between the near-ambient 1.6 GPa structure (I) and the 29.5 GPa high-pressure structure (LGR), both reported by Sun et al. as *.cif* files in their original publication [4].

A. Extracting octahedra from $La_3Ni_2O_7$

1. *Generalized octahedral parametrization*

We first generalize the model described in [35], to include octahedral non-collinear apical distortions, which are present in the (I) structure of $La_3Ni_2O_7$. To that end, P2 in [35] becomes[1]:

$$\mathbf{F} = \begin{pmatrix} 1 & 0 & 0 \\ 0 & 1 & 0 \\ f_{31} & f_{32} & f_{33} \end{pmatrix}, \tag{1}$$

with f_{31}, f_{32}, and f_{33} real parameters. With this generalization, the centroid of an octahedron in P12 is now given by:

$$\mathbf{C} = \frac{1}{6}\sum_{n} \mathbf{O}^{n} = \frac{\hat{k}(\mathbb{I} - \mathbf{F})\mathbf{P}}{6} + \mathbf{D}. \tag{2}$$

2. *Geometric interpretation of the* **F***-matrix*

The **F**-matrix in Eq. 1 contains the information of the magnitude and collinearity of the octahedral apical distortion. To see this, we write the first matrix on the right-hand side

[1] We will employ the notation P# to refer to equations in [35], not to confuse them with equations here.

of P1 like:

$$\begin{pmatrix} \mathbb{I} \\ -\mathbf{F} \end{pmatrix} = \begin{pmatrix} \hat{i} \\ \hat{j} \\ \hat{k} \\ -\hat{i} \\ -\hat{j} \\ -\mathbf{f} \end{pmatrix},$$

where we have used Eq. 1 and defined $\mathbf{f} = \begin{pmatrix} f_{31} & f_{32} & f_{33} \end{pmatrix}$. This represents the corners of a regular, orthogonal octahedron of unitary arm lengths, except that the $-k$ corner has been changed from $-\hat{k}$ to $-\mathbf{f}$. The corners $\{\pm i, \pm j\}$ are clearly centrosymmetric, but the corners $\{\pm k\}$ will be only if $\mathbf{f} = \hat{k}$. The dot products between the i, j and k corners and the $-k$ corner are:

$$\hat{i} \cdot -\mathbf{f} = -f_{31} = |\mathbf{f}| \cos \xi_1,$$

$$\hat{j} \cdot -\mathbf{f} = -f_{32} = |\mathbf{f}| \cos \xi_2,$$

$$\hat{k} \cdot -\mathbf{f} = -f_{33} = |\mathbf{f}| \cos \xi_3.$$

If $f_{31} = f_{32} = 0$, then $|f| = f_{33}$, $\xi_1 = 90°$, $\xi_2 = 90°$ and $\xi_3 = 180°$. That is, the apical distortion is collinear. If $|f| = 1$, the $-k$ corner has unitary arm length, but the collinearity will depend on the values of f_{31}, f_{32} and f_{33}. This suggest a decomposition of the $\mathbf{F}$-matrix into a $\mathbf{F}_L$-matrix carrying the magnitude of the apical distortion and a $\mathbf{F}_G$-matrix encoding unitary non-collinear distortions:

$$\mathbf{F} = \mathbf{F}_L \mathbf{F}_G = \begin{pmatrix} 1 & 0 & 0 \\ 0 & 1 & 0 \\ 0 & 0 & |\mathbf{f}| \end{pmatrix} \begin{pmatrix} 1 & 0 & 0 \\ 0 & 1 & 0 \\ f_{31}/|\mathbf{f}| & f_{32}/|\mathbf{f}| & f_{33}/|\mathbf{f}| \end{pmatrix}.$$

3. Extracting parameters from a single octahedron

Following the $\mathbf{F}$-matrix generalization in Eq. 1, the algorithm to extract parameters from a single octahedron in Section 1.3 of [35] is rewritten follows:

From P1, we begin by splitting the known $\mathbf{O}$-matrix as:

$$\mathbf{O}^{+} = \mathbf{P} + 1_3\mathbf{D},$$

$$\mathbf{O}^{-} = -\mathbf{F}\mathbf{P} + 1_3\mathbf{D},$$

where $1_3 = \begin{pmatrix} 1 & 1 & 1 \end{pmatrix}^T$. Then, we define $\mathbf{O}_g$, $\mathbf{W}$ and $\mathbf{W}_g$, such that:

$$\mathbf{O}_g = \mathbf{O}^{+} - \mathbf{O}^{-} = (\mathbb{I} + \mathbf{F})\mathbf{LGR} = (\mathbb{I} + \mathbf{F})\mathbf{WR} = \mathbf{W}_g\mathbf{R}. \quad (3)$$

$\mathbf{O}_g$ is simply the product of the lower triangular matrix $\mathbf{W}_g$ (see Eqs. 1, P4 and P5) and $\mathbf{R} \in SO(3)$: therefore, we can apply the so-called QR-decomposition method on $\mathbf{O}_g^T$ [36], such that[2]:

$$\begin{aligned} \mathbf{O}_g^T &= \mathbf{R}^T\mathbf{W}_g^T \Rightarrow \\ \mathbf{O}_g &= \mathbf{W}_g\mathbf{R}, \end{aligned}$$

consistent with Eq. 3. Thus, $\mathbf{W}_g$ and $\mathbf{R}$ are known and we can directly extract the rotation angles from $\mathbf{R}$ using Eqs. P6 - P8. Moreover, using Eq. 2 we can show that:

$$\mathbf{O}^{-} - 1_3\mathbf{C} = \frac{1}{6}\begin{pmatrix} f_{31} - 6 & f_{32} & f_{33} - 1 \\ f_{31} & f_{32} - 6 & f_{33} - 1 \\ -5f_{31} & -5f_{32} & -(5f_{33} + 1) \end{pmatrix}\mathbf{P},$$

[2] The method decomposes a square matrix into a product of an element of $SO(3)$ and an upper triangular matrix.

$$\mathbf{O}^{+} - 1_3\mathbf{C} = \frac{1}{6}\begin{pmatrix} f_{31}+6 & f_{32} & f_{33}-1 \\ f_{31} & f_{32}+6 & f_{33}-1 \\ f_{31} & f_{32} & f_{33}+5 \end{pmatrix}\mathbf{P},$$

and define:

$$\mathbf{O}_c = (\mathbf{O}^{-} - 1_3\mathbf{C})(\mathbf{O}^{+} - 1_3\mathbf{C})^{-1}$$

$$= \frac{1}{f_{31}+f_{32}+f_{33}+5}\times$$

$$\begin{pmatrix} f_{31}-f_{32}-f_{33}-5 & 2f_{32} & 2(f_{33}-1) \\ 2f_{31} & -f_{31}+f_{32}-f_{33}-5 & 2(f_{33}-1) \\ -4f_{31} & -4f_{32} & -f_{31}-f_{32}-5f_{33}-1 \end{pmatrix}, \tag{4}$$

thus:

$$f_{33} = \begin{cases} \frac{x-5}{x+1} & \text{where } x = \frac{\mathbf{O}_c^{11}+\mathbf{O}_c^{22}}{\mathbf{O}_c^{13}} \text{ if } \mathbf{O}_c^{13} \neq 0 \\ 1 & \text{if } \mathbf{O}_c^{13} = 0 \end{cases},$$

$$f_{31} = \begin{cases} (f_{33}-1)\frac{\mathbf{O}_c^{21}}{\mathbf{O}_c^{13}} & \text{if } \mathbf{O}_c^{13} \neq 0 \\ \frac{6\mathbf{O}_c^{21}}{2-\mathbf{O}_c^{12}-\mathbf{O}_c^{21}} & \text{if } \mathbf{O}_c^{13} = 0 \end{cases},$$

$$f_{32} = \begin{cases} (f_{33}-1)\frac{\mathbf{O}_c^{12}}{\mathbf{O}_c^{13}} & \text{if } \mathbf{O}_c^{13} \neq 0 \\ \frac{6\mathbf{O}_c^{12}}{2-\mathbf{O}_c^{12}-\mathbf{O}_c^{21}} & \text{if } \mathbf{O}_c^{13} = 0 \end{cases}, \tag{5}$$

which determines the $\mathbf{F}$-matrix. The centroid $\mathbf{C}$ can be directly extracted from the $\mathbf{O}$-matrix using the first equality in Eq. 2, and therefore the $\mathbf{O}_c$-matrix is known. If the $\mathbf{F}$-matrix calculated from the $\mathbf{O}_c$-matrix via Eq. 5 yields a different $\mathbf{O}_c$-matrix when plugged into Eq. 4, the $\mathbf{O}$-matrix under consideration is beyond the model in Eq. P1. Finally, we compute:

$$\mathbf{W} = (\mathbb{I} + \mathbf{F})^{-1}\mathbf{W}_g,$$

which can be written as:

$$\mathbf{W} = \begin{pmatrix} \mathbf{W}_1 \\ \mathbf{W}_2 \\ \mathbf{W}_3, \end{pmatrix},$$

where each row is an octahedron arm vector. From Eq. P5, we extract the angles $\{\alpha', \beta', \gamma'\}$ between the arm vectors using their dot products. Also, the arm lengths satisfy $a' = |\mathbf{W}_1|$, $b' = |\mathbf{W}_2|$ and $c' = |\mathbf{W}_3|$. Thus, we have determined the **L**- and **G**-matrices.

For completion, we define the off-centering vector **d** as the difference between the position **Q** of the caged atom in the crystal structure and the centroid of the octahedron given by Eq. 2:

$$\mathbf{d} = \mathbf{Q} - \mathbf{C}.$$

4. Obtaining the octahedral parameters

The algorithm described in Section IX A 3 above is employed to extract the octahedral parameters in the unit cells of (I) and (LGR). Taking the unit cell of (I), for example (see Fig. S18), one notices the existence of four inequivalent octahedra within each bilayer, where the blue-green and the red-yellow pairs are each forming bipartite two-dimensional lattices. These sublattices are described exactly by the methods in [35], however, here we must guarantee that both layers remain interconnected, representing a three-dimensional condition suggested but not treated in [35]. To address this, we make use of the mirror plane between the layers: we parametrize the bottom layer using P1 with our generalized **F**-matrix, but for the top layer we define:

$$\mathbf{O} = \begin{pmatrix} \mathbf{F} \\ -\mathbb{I} \end{pmatrix} \mathbf{P} + 1_6\mathbf{D}, \tag{6}$$

which essentially allows us to treat the octahedral coordinates symmetrically along the c-axis with respect to the mirror plane. Of course, the algorithm described in Section IX A 3 needs to be modified for this definition of the **O**-matrix, but this is quite simple by just

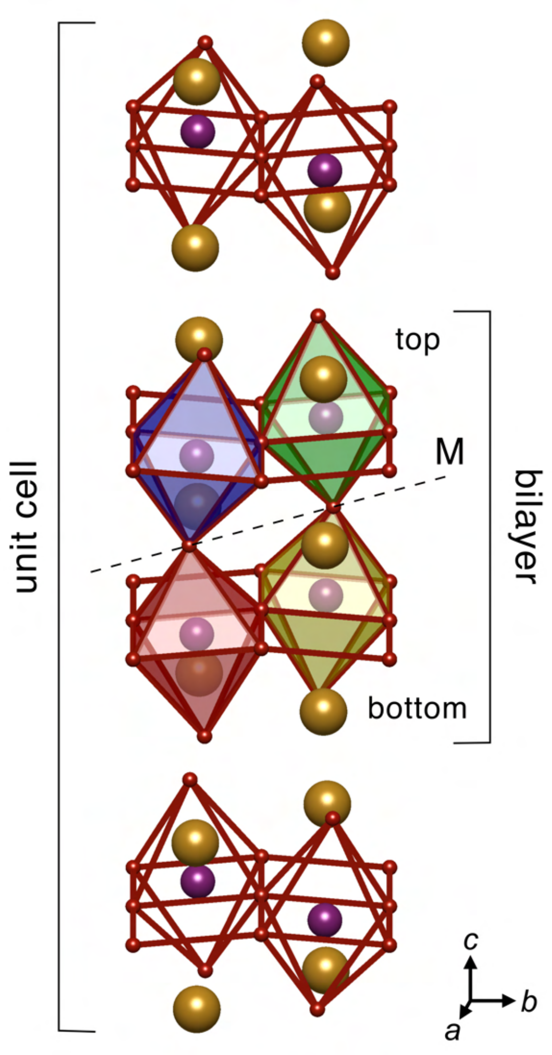

FIG. S18. The unit cell of (I), and of $La_3Ni_2O_7$ in general, is formed by stacking equivalent bilayers along the c-axis with a relative in-plane shift of $[1/2, 0, 0]$. There are four inequivalent Ni-sites within a bilayer, which can be understood as a pair of interconnected two-dimensional layers of corner-sharing-octahedra, each hosting two inequivalent Ni-sites (here blue-green and red-yellow). A mirror plane M is located where the two layers interconnect.

following the procedure in Section IX A 3.

The octahedral parameters corresponding to the four full structures examined in the main text are compiled in Fig. S19.

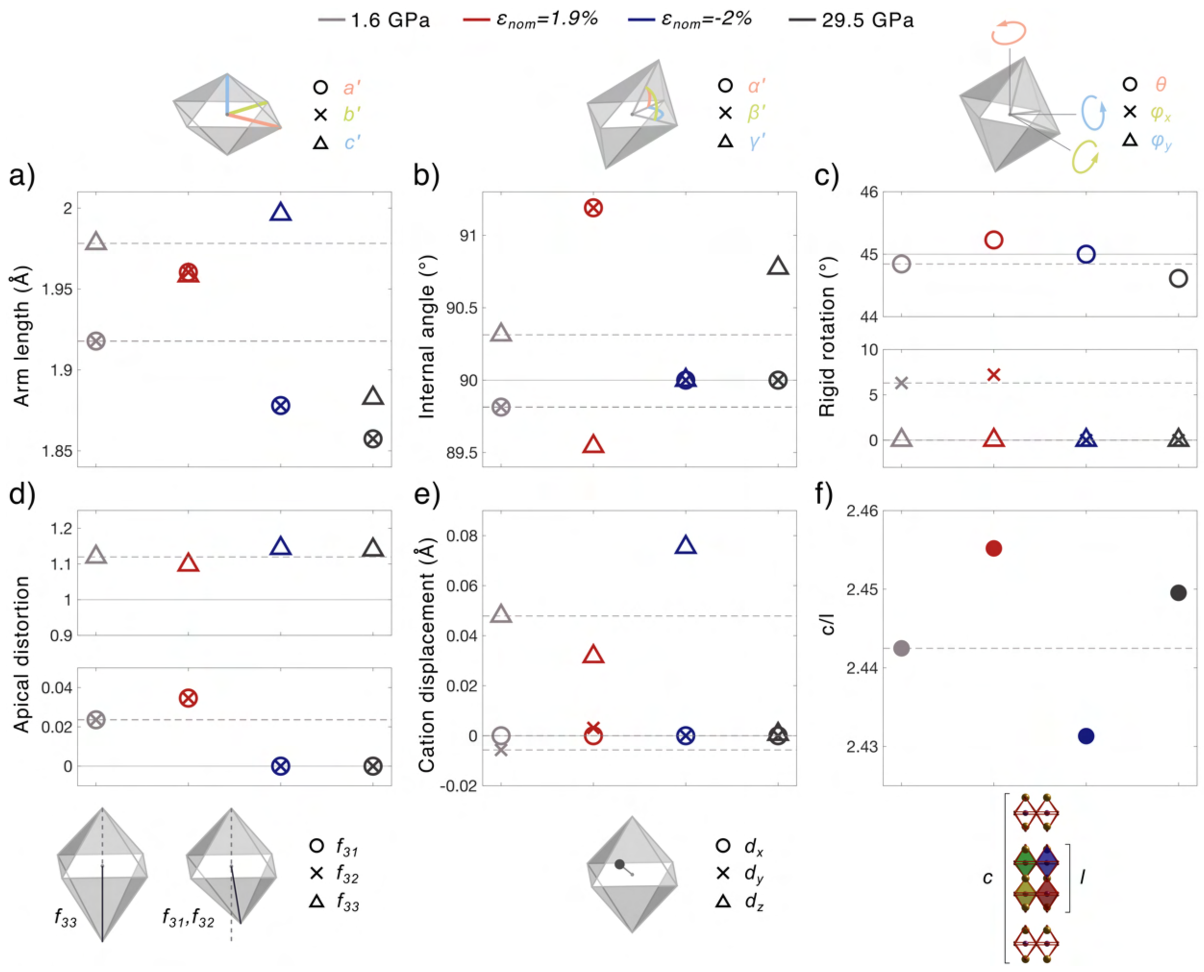

FIG. S19. Octahedral parameters for the near-ambient 1.6 GPa structure and the structures for epitaxial tensile strain on STO; epitaxial compressive strain on SLAO; and the 29.5 GPa high-pressure structure reported by [4], respectively. A schematic is used to visually represent the parameters in each case. **a)** Octahedral arm-lengths. **b)** Octahedral internal angles. **c)** Octahedral rigid rotations. **d)** Octahedral apical distortions. **e)** Cation displacement from octahedron centroid. **f)** Ratio between c-axis length and bilayer thickness l. The value remains relatively unchanged for all the structures within about 1%.

B. Decomposing the straining process

1. Reconstructing the bipartite layers

Once we have the octahedral parameters for the (I) and (LGR) structures, they are input to the main octahedron algorithm described in Section 1.11 in [35], modified with all the generalizations mentioned above. The algorithm reconstructs the top and bottom bipartite layers in the specific structure and outputs the **P**-, **L**-, **G**-, **R**-, $\mathbf{F}_L$- and $\mathbf{F}_G$-matrices at each octahedral site.

2. *Layer distortion decomposition*

The sets of matrices obtained in the previous step for the (I) and (LGR) bipartite layers are used to generate new sets of bipartite structures (referred to as partial structures) which carry only specific distortions resulting from the straining process, as described in Section 2 in [35]. For example, if the desired partial structure contains only the rotational changes, the **R**-matrix from the (LGR) configuration is used, while the remaining matrices defining the structure are taken from the (I) geometry. In this case, the partial structure is referred to as (R).

3. *Reconstruct $La_3Ni_2O_7$*

The layers embodying the specific distortions are connected into bilayers, and the bilayers are stacked accordingly to recover the full structure of $La_3Ni_2O_7$. Our definition in Eq. 6 is satisfactory, as all the top and bottom bipartite layers display perfect connectivity, no matter the specific distortion being considered. The generated $La_3Ni_2O_7$ partial structures have a unit cell that is fully constrained in-plane, as well as fixed octahedral geometry, that is, fixed O positions; however, the out-of-plane lattice parameter is unknown because the spacing between two bilayers forming the $La_3Ni_2O_7$ unit cell (non-corner sharing) is unconstrained in our model. Furthermore, the La and Ni positions will likely react to the particular octahedral geometry. We therefore aim for a relaxation of the partial structures that allows for out-of-plane lattice parameter variation and free Ni and La movement, while keeping the octahedral geometry of both bilayers unchanged. To our knowledge, implementing relaxations which constrain the absolute O positions while allowing unit cell changes is not straightforward within widely available packages such as QUANTUM ESPRESSO, VASP and CP2K. For this work, we find that the ratio between c-axis length and bilayer thickness remains relatively unchanged between the (I) and (LGR) $La_3Ni_2O_7$ structures (see Fig. S18). For our models, we therefore interpolate the out-of-plane lattice parameter for the partial structures and perform a simple relaxation with the unit cell fixed and free Ni and La positions. As a test for the whole protocol, the (I) and (LGR) structures generated from the decomposition algorithm recover the full $La_3Ni_2O_7$ structures exactly.

X. TRANSFORMATION INTO THE LOCAL BASIS

The orbital-projected wavefunctions of the Ni 3d states were transformed into the local octahedral basis using the Wigner rotation formalism [37]. At each Ni site, the **R**-matrix was computed following the procedure outlined in Section IX A 3, and used to construct the corresponding Wigner D-matrix for angular momentum $l = 2$. The complex coefficients of the projected wavefunctions were then rotated via the D-matrix, and their squared norms were evaluated to obtain the corresponding orbital weights.

XI. BILAYER VS COMPLETE STRUCTURE

To enable faster computational screening, initial calculations were performed on single bilayers instead of the complete Ruddlesden-Popper unit cell. Calculations on $La_3Ni_2O_7$ bilayers extracted directly from the strain-decomposed structures described above are presented in Fig. S20, comparing the results for the near-ambient and high-pressure bulk structures. These bilayer results reproduce the same trends and behavior observed in complete unit cell calculations, with the exception of band doubling effects arising from the staggered bilayer stacking in the $La_3Ni_2O_7$ unit cell.

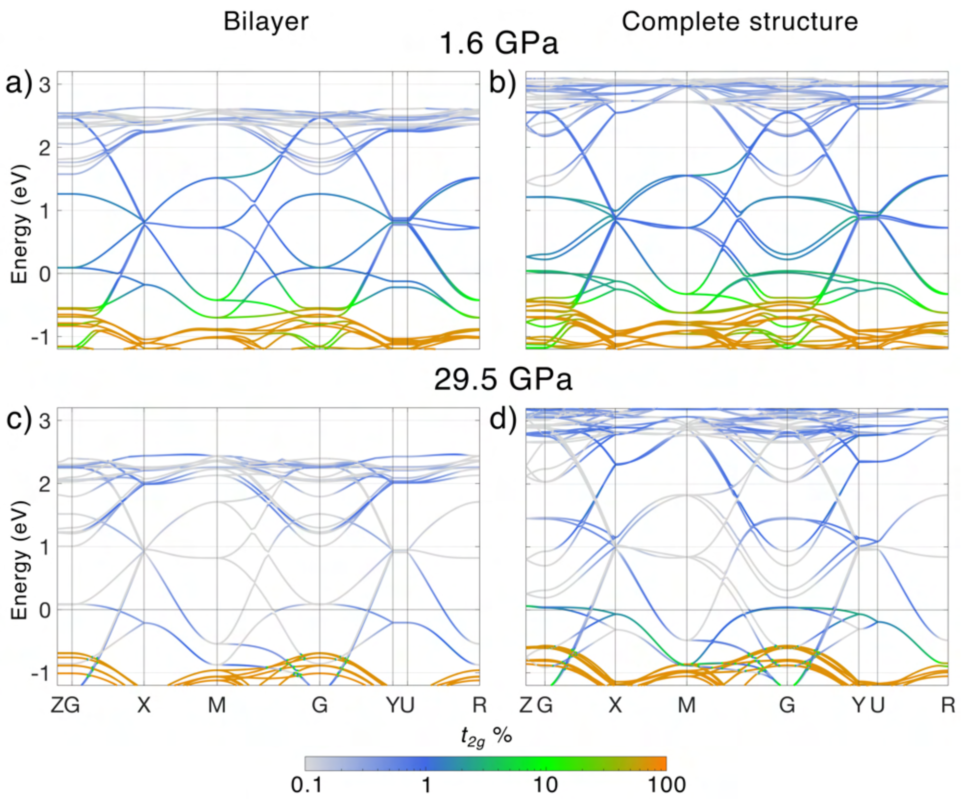

FIG. S20. Comparison between low-energy band structures of $La_3Ni_2O_7$ without Hubbard U projected to the local t_{2g} manifold of Ni 3d states for the near-ambient 1.6 GPA structure (upper row) and the 29.5 GPa high-pressure structure reported by [4] (lower row). **a, c)** Single bilayer calculations. **b, d)** Complete structure calculations.

XII. GGA WITH AND WITHOUT HUBBARD U

Comparison of Figs. S21 and S22 shows that the most prominent effect of the Hubbard U on the band structure is the shift of the Ni 3d t_{2g}-derived states towards higher binding energies. This shift is crucial for reducing the contribution of t_{2g} states to the low-energy electronic structure in configurations without octahedral tilts. While the rotational distortions alone already diminish the presence of t_{2g} in the unoccupied states, as seen in Figs. S22d,e, it is only upon introducing electron correlations that the residual t_{2g} weight near the Fermi level is largely suppressed, as observed in Figs. S21d,e. In contrast, the increased octahedral tilting present in the structure shown in Fig. S22c leads to enhanced t_{2g} hybridization, and in this case, the Hubbard U has no effect in suppressing it, as seen in Fig. S21c. Interestingly, the reduction of the t_{2g} contribution near the Fermi level is more pronounced in the high-pressure structure than in the structure under epitaxial compressive strain, the

latter exhibiting a superconducting T_c approximately half that of the former [4, 12]. This suggests that a residual Ni 3d t_{2g} contribution may be detrimental to superconductivity and motivates further theoretical investigation into the role of these orbitals in the low-energy structure of $La_3Ni_2O_7$.

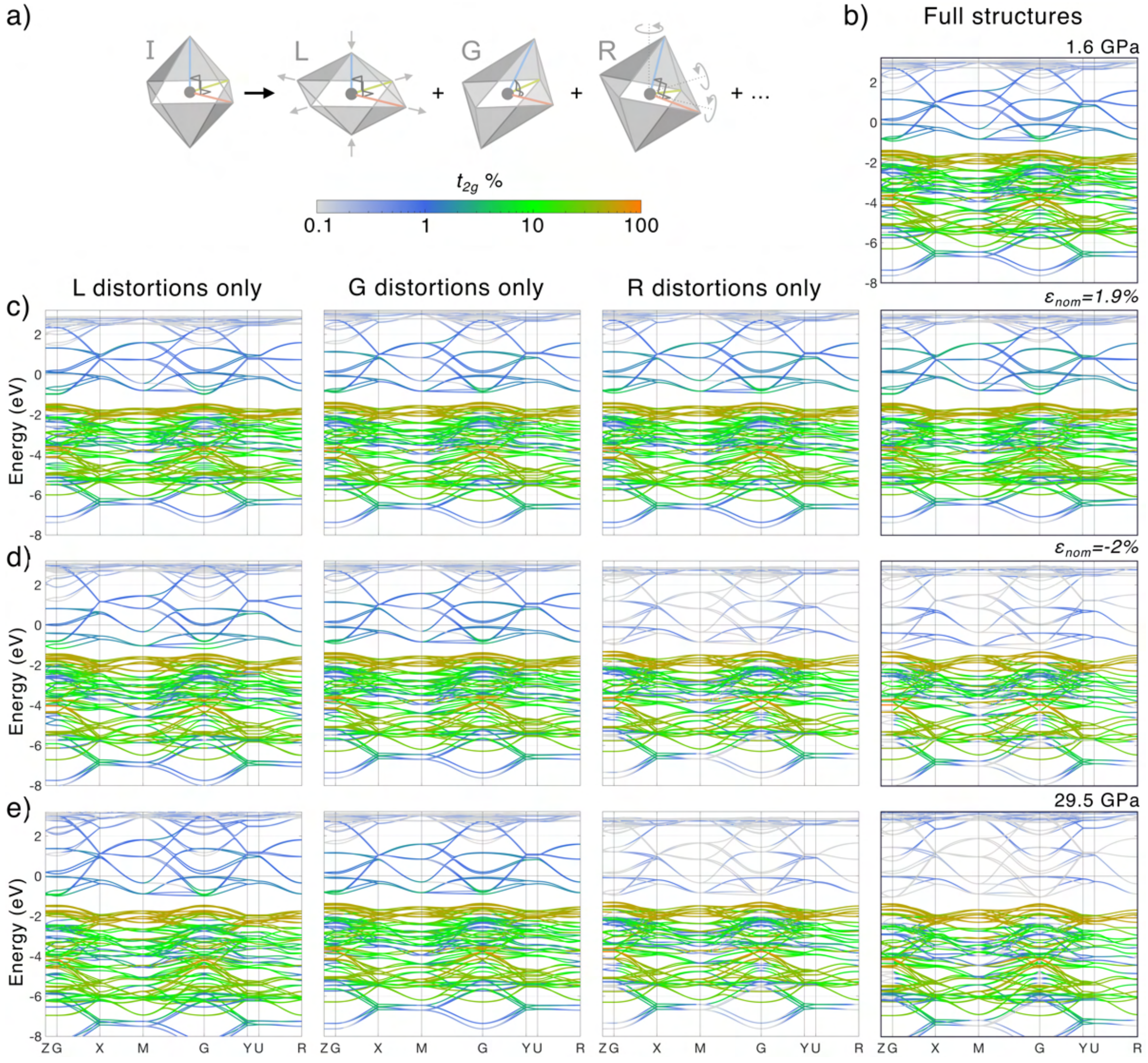

FIG. S21. Octahedral distortion-decomposed electronic structure calculations of $La_3Ni_2O_7$ with $U = 4$ eV on the Ni site. **a)** Schematic representation of different distortions applied to a regular octahedron (I), including bond length changes (L), internal angle changes (G), and rigid rotations (R). The deformation of a system of corner-sharing octahedra can be decomposed into partial structures embodying the independent distortions. **b-e)** Low-energy band structures of $La_3Ni_2O_7$ projected to the local t_{2g} manifold of Ni $3d$ states. (b) Near-ambient 1.6 GPa structure. (c-e) The L, G and R partial structures obtained from decomposing the strain between the near-ambient 1.6 GPa structure and the structures for (c) epitaxial tensile strain on STO; (d) epitaxial compressive strain on SLAO; and (e) the 29.5 GPa high-pressure structure reported by [4]. The energy and hybridization range has been extended with respect to the main text to show the complete t_{2g} manifold.

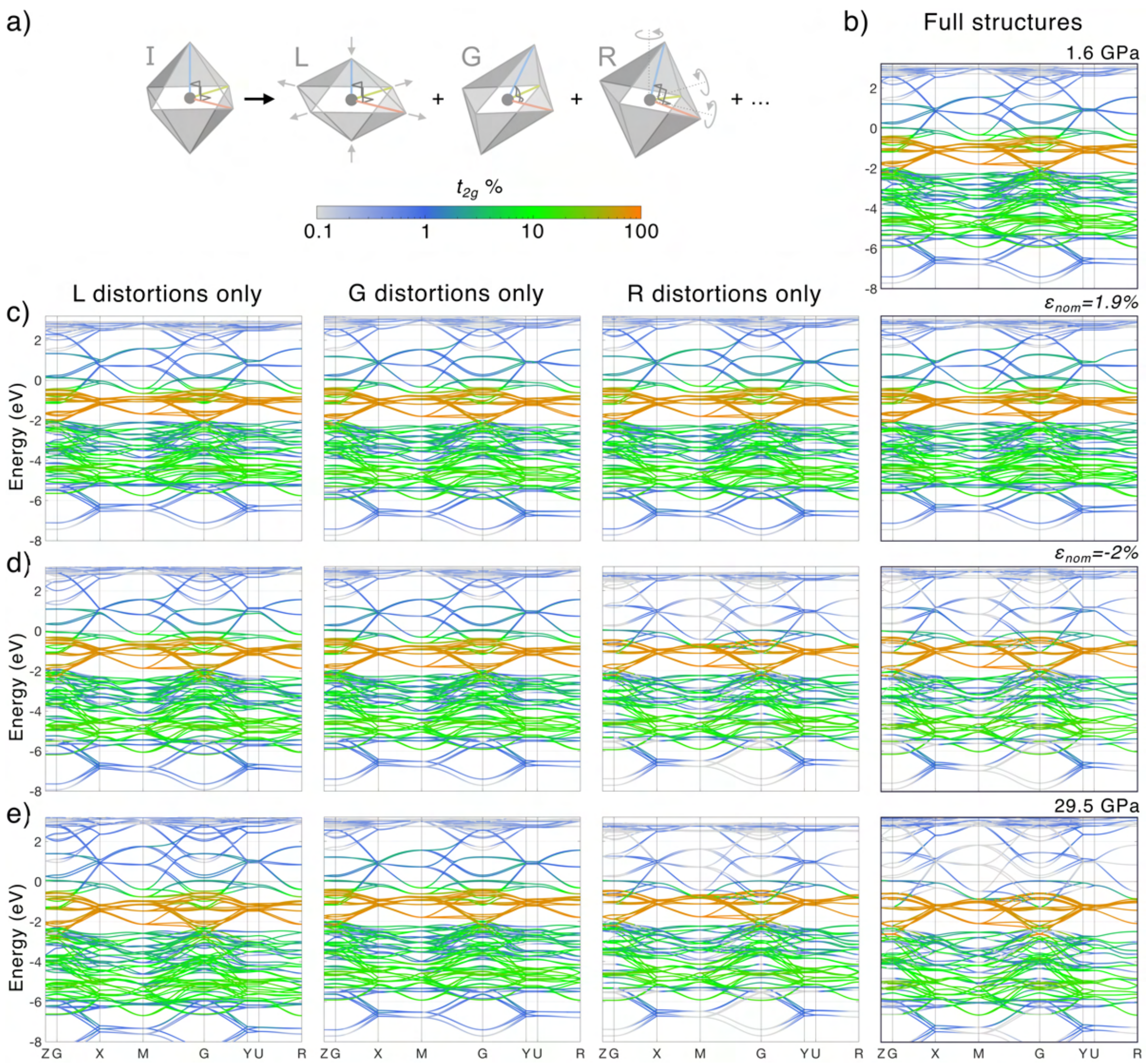

FIG. S22. Same as Fig. S21 but without Hubbard U.

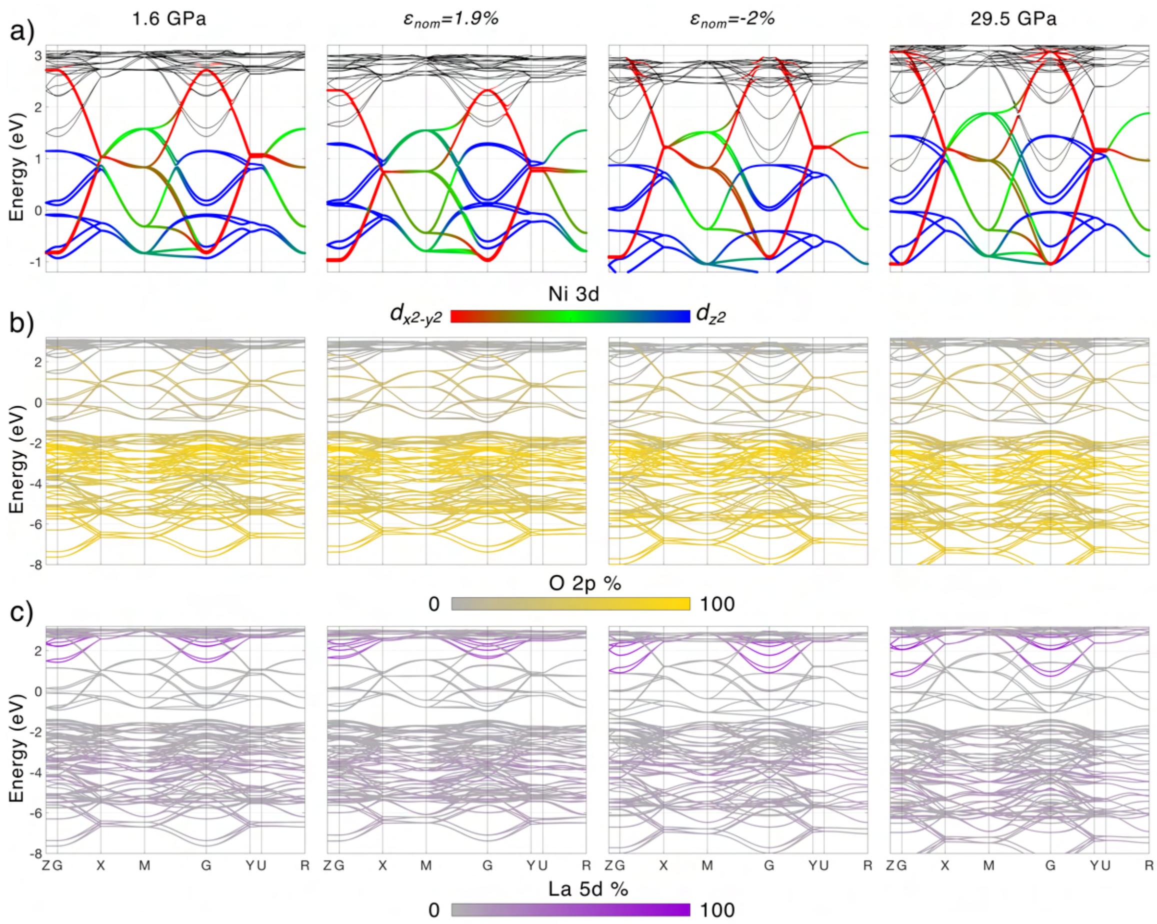

FIG. S23. Additional projected band structures of $La_3Ni_2O_7$ for the near-ambient 1.6 GPa structure and the structures for epitaxial tensile strain on STO; epitaxial compressive strain on SLAO; and the 29.5 GPa high-pressure structure reported by [4], respectively. **a)** Polarization plot showing the local projection to $d_{x^2-y^2}$ and d_{z^2} Ni 3d states. The line thickness is proportional to the orbital weight contribution of the states relative to the total. Lines are rendered in black when this contribution is less than 3%. **b)** Projection to O 2p states. **c)** Projection to La 5d states.

[1] Y. Jiang, Z. Chen, Y. Han, P. Deb, H. Gao, S. Xie, P. Purohit, M. W. Tate, J. Park, S. M. Gruner, V. Elser, and D. A. Muller, Electron ptychography of 2D materials to deep sub-Ångström resolution, Nature **559**, 343 (2018).

[2] Z. Chen, Y. Jiang, Y.-T. Shao, M. E. Holtz, M. Odstrčil, M. Guizar-Sicairos, I. Hanke, S. Ganschow, D. G. Schlom, and D. A. Muller, Electron ptychography achieves atomic-resolution limits set by lattice vibrations, Science **372**, 826 (2021).

[3] H. T. Philipp, M. W. Tate, K. S. Shanks, L. Mele, M. Peemen, P. Dona, R. Hartong, G. van Veen, Y.-T. Shao, Z. Chen, J. Thom-Levy, D. A. Muller, and S. M. Gruner, Very-high dynamic range, 10,000 frames/second pixel array detector for electron microscopy, Microscopy and Microanalysis **28** (2022).

[4] H. Sun, M. Huo, X. Hu, J. Li, Z. Liu, Y. Han, L. Tang, Z. Mao, P. Yang, B. Wang, J. Cheng, D.-X. Yao, G.-M. Zhang, and M. Wang, Signatures of superconductivity near 80 K in a nickelate under high pressure, Nature **621**, 493–498 (2023).

[5] Z. Dong, M. Huo, J. Li, J. Li, P. Li, H. Sun, Y. Lu, M. Wang, Y. Wang, and Z. Chen, Visualization of oxygen vacancies and self-doped ligand holes in $La_3Ni_2O_{7-\delta}$, Nature **630**, 847 (2023).

[6] P. Thibault and M. Guizar-Sicairos, Maximum-likelihood refinement for coherent diffractive imaging, New Journal of Physics **14**, 063004 (2012).

[7] K. Wakonig, H.-C. Stadler, M. Odstrčil, E. H. Tsai, A. Diaz, M. Holler, I. Usov, J. Raabe, A. Menzel, and M. Guizar-Sicairos, Ptychoshelves, a versatile high-level framework for high-performance analysis of ptychographic data, Journal of Applied Crystallography **53**, 574 (2020).

[8] H. KP, R. Xu, K. Patel, K. J. Crust, A. Khandelwal, C. Zhang, S. Prosandeev, H. Zhou, Y.-T. Shao, L. Bellaiche, H. Y. Hwang, and D. A. Muller, Electron ptychography reveals a ferroelectricity dominated by anion displacements, Nature Materials **24**, 1433–1440 (2025).

[9] S. Karapetyan, S. E. Zeltmann, G. Wilk, T.-K. Chen, V. D.-H. Hou, and D. A. Muller, Nature Communications (2025).

[10] D. Ferenc Segedin, B. H. Goodge, G. A. Pan, Q. Song, H. LaBollita, M.-C. Jung, H. El-Sherif, S. Doyle, A. Turkiewicz, N. K. Taylor, J. A. Mason, A. T. N'Diaye, H. Paik, I. E. Baggari, A. S.

Botana, L. F. Kourkoutis, C. M. Brooks, and J. A. Mundy, Limits to the strain engineering of layered square-planar nickelate thin films, Nature Communications **14**, 1468 (2023).

[11] T. Cui, S. Choi, T. Lin, C. Liu, G. Wang, N. Wang, S. Chen, H. Hong, D. Rong, Q. Wang, Q. Jin, J.-O. Wang, L. Gu, C. Ge, C. Wang, J.-G. Cheng, Q. Zhang, L. Si, K.-J. Jin, and E.-J. Guo, Strain-mediated phase crossover in Ruddlesden–Popper nickelates, Communications Materials **5**, 32 (2024).

[12] E. K. Ko, Y. Yu, Y. Liu, L. Bhatt, J. Li, V. Thampy, C.-T. Kuo, B. Y. Wang, Y. Lee, K. Lee, J.-S. Lee, B. H. Goodge, D. A. Muller, and H. Y. Hwang, Signatures of ambient pressure superconductivity in thin film $La_3Ni_2O_7$, Nature **638**, 935–940 (2025).

[13] Y. Liu, E. K. Ko, Y. Tarn, L. Bhatt, J. Li, V. Thampy, B. H. Goodge, D. A. Muller, S. Raghu, Y. Yu, and H. Y. Hwang, Superconductivity and normal-state transport in compressively strained $La_2PrNi_2O_7$ thin films, Nature Materials **24**, 1221–1227 (2025).

[14] Y. Zhu, D. Peng, E. Zhang, B. Pan, X. Chen, L. Chen, H. Ren, F. Liu, Y. Hao, N. Li, Z. Xing, F. Lan, J. Han, J. Wang, D. Jia, H. Wo, Y. Gu, Y. Gu, L. Ji, W. Wang, H. Gou, Y. Shen, T. Ying, X. Chen, W. Yang, H. Cao, C. Zheng, Q. Zeng, J.-g. Guo, and J. Zhao, Superconductivity in pressurized trilayer $La_4Ni_3O_{10-\delta}$ single crystals, Nature **631**, 531–536 (2024).

[15] H. Wang, H. Huang, G. Zhou, W. Lv, C. Yue, L. Xu, X. Wu, Z. Nie, Y. Chen, Y.-J. Sun, W. Chen, H. Yuan, Z. Chen, and Q.-K. Xue, Electronic structures across the superconductor-insulator transition at $La_{2.85}Pr_{0.15}Ni_2O_7/SrLaAlO_4$ interfaces, arXiv preprint arXiv:2502.18068 (2025).

[16] M. Osada, C. Terakura, A. Kikkawa, M. Nakajima, H.-Y. Chen, Y. Nomura, Y. Tokura, and A. Tsukazaki, Strain-tuning for superconductivity in $La_3Ni_2O_7$ thin films, Communications Physics **8** (2025).

[17] B. Geisler, J. J. Hamlin, G. R. Stewart, R. G. Hennig, and P. Hirschfeld, Electronic reconstruction and interface engineering of emergent spin fluctuations in compressively strained $La_3Ni_2O_7/SrLaAlO_4$ (001), arXiv preprint arXiv:2503.10902 (2025).

[18] C. Le, J. Zhan, X. Wu, and J. Hu, Opposite-mirror-parity scattering as the origin of superconductivity in strained bilayer nickelates, arXiv preprint arXiv:2501.14665 (2025).

[19] P. Puphal, P. Reiss, N. Enderlein, Y.-M. Wu, G. Khaliullin, V. Sundaramurthy, T. Priessnitz, M. Knauft, A. Suthar, L. Richter, M. Isobe, P. A. van Aken, H. Takagi, B. Keimer, Y. E.

Suyolcu, B. Wehinger, P. Hansmann, and M. Hepting, Unconventional crystal structure of the high-pressure superconductor $La_3Ni_2O_7$, Physical Review Letters **133**, 146002 (2024).

[20] X. Chen, J. Zhang, A. S. Thind, S. Sharma, H. LaBollita, G. Peterson, H. Zheng, D. P. Phelan, A. S. Botana, R. F. Klie, and J. F. Mitchell, Polymorphism in the Ruddlesden–Popper nickelate $La_3Ni_2O_7$: discovery of a hidden phase with distinctive layer stacking, Journal of the American Chemical Society **146**, 3640 (2024).

[21] H. Wang, L. Chen, A. Rutherford, H. Zhou, and W. Xie, Long-range structural order in a hidden phase of Ruddlesden–Popper bilayer nickelate $La_3Ni_2O_7$, Inorganic Chemistry **63**, 5020 (2024).

[22] F. Li, N. Guo, Q. Zheng, Y. Shen, S. Wang, Q. Cui, C. Liu, S. Wang, X. Tao, G.-M. Zhang, and J. Zhang, Design and synthesis of three-dimensional hybrid Ruddlesden-Popper nickelate single crystals, Physical Review Materials **8**, 053401 (2024).

[23] Z. Zhang, M. Greenblatt, and J. Goodenough, Synthesis, structure, and properties of the layered perovskite $La_3Ni_2O_{7-\delta}$, Journal of Solid State Chemistry **108**, 402 (1994).

[24] S. Taniguchi, T. Nishikawa, Y. Yasui, Y. Kobayashi, J. Takeda, S.-I. Shamoto, and M. Sato, Transport, magnetic and thermal properties of $La_3Ni_2O_{7-\delta}$, Journal of the Physical Society of Japan **64**, 1644 (1995).

[25] Y. Kobayashi, S. Taniguchi, M. Kasai, M. Sato, T. Nishioka, and M. Kontani, Transport and magnetic properties of $La_3Ni_2O_{7-\delta}$ and $La_4Ni_3O_{10-\delta}$, Journal of the Physical Society of Japan **65**, 3978 (1996).

[26] G. Zhou, W. Lv, H. Wang, Z. Nie, Y. Chen, Y. Li, H. Huang, W. Chen, Y. Sun, Q.-K. Xue, and Z. Chen, Ambient-pressure superconductivity onset above 40 K in $(La,Pr)_3Ni_2O_7$ films films, Nature **640**, 641 (2025).

[27] L. F. Kourkoutis, H. Xin, T. Higuchi, Y. Hotta, J. Lee, Y. Hikita, D. Schlom, H. Hwang, and D. Muller, Atomic-resolution spectroscopic imaging of oxide interfaces, Philosophical Magazine **90**, 4731 (2010).

[28] B. H. Goodge, D. Li, K. Lee, M. Osada, B. Y. Wang, G. A. Sawatzky, H. Y. Hwang, and L. F. Kourkoutis, Doping evolution of the Mott–Hubbard landscape in infinite-layer nickelates, Proceedings of the National Academy of Sciences **118** (2021).

[29] B. Y. Wang, Y. Zhong, S. Abadi, Y. Liu, Y. Yu, X. Zhang, Y.-M. Wu, R. Wang, J. Li, Y. Tarn, E. K. Ko, V. Thampy, M. Hashimoto, D. Lu, Y. S. Lee, T. P. Devereaux, C. Jia, H. Y. Hwang,

and Z.-X. Shen, Electronic structure of compressively strained thin film $La_2PrNi_2O_7$, arXiv preprint arXiv:2504.16372 (2025).

[30] G. Zhou, H. Wang, H. Huang, Y. Chen, F. Peng, W. Lv, Z. Nie, W. Wang, Q. Xue, and Z. Chen, Superconductivity onset above 60 K in ambient-pressure nickelate films, arXiv preprint arXiv:2512.04708 (2025).

[31] J. B. Nelson and D. P. Riley, An experimental investigation of extrapolation methods in the derivation of accurate unit-cell dimensions of crystals, Proceedings of the Physical Society **57**, 160 (1945).

[32] H. Wang, L. Chen, A. Rutherford, H. Zhou, and W. Xie, Long-range structural order in a hidden phase of Ruddlesden–Popper bilayer nickelate $La_2PrNi_2O_7$, Inorganic Chemistry **63**, 5020 (2024).

[33] G. Wang, N. N. Wang, X. L. Shen, J. Hou, L. Ma, L. F. Shi, Z. A. Ren, Y. D. Gu, H. M. Ma, P. T. Yang, Z. Y. Liu, H. Z. Guo, J. P. Sun, G. M. Zhang, S. Calder, J.-Q. Yan, B. S. Wang, Y. Uwatoko, and J.-G. Cheng, Pressure-induced superconductivity in polycrystalline $La_2PrNi_2O_{7-\delta}$, Physical Review X **14**, 011040 (2024).

[34] L. Wang, Y. Li, S.-Y. Xie, F. Liu, H. Sun, C. Huang, Y. Gao, T. Nakagawa, B. Fu, B. Dong, Z. Cao, R. Yu, S. I. Kawaguchi, H. Kadobayashi, M. Wang, C. Jin, H. kwang Mao, and H. Liu, Structure responsible for the superconducting state in $La_3Ni_2O_7$ at high-pressure and low-temperature conditions, Journal of the American Chemical Society **146**, 7506 (2024).

[35] E. Abarca Morales, The 4-octahedra model, Frontiers in Materials **11**, 1461579 (2024).

[36] W. Gander, Algorithms for the QR decomposition, Res. Rep **80**, 1251 (1980).

[37] J. J. Sakurai and J. Napolitano, *Modern quantum mechanics* (Cambridge University Press, 2020).
